# Targeting the exosomal CaMK2A-ZDHHC3-GPX4 pathway reprograms tumor-associated macrophages and enhances anti-PD-1/PD-L1 immunotherapy in gastric cancer

**DOI:** 10.7150/ijbs.128976

**Published:** 2026-05-18

**Authors:** Zetian Chen, Hongxin Huang, Yikai Shen, Masami Yamamoto, Tetsuya Tsukamoto, Sachiyo Nomura, Tianlu Jiang, Zekuan Xu, Zheng Li

**Affiliations:** 1 Gastric Cancer Center, Department of General Surgery, The First Affiliated Hospital with Nanjing Medical University, Nanjing, Jiangsu, China.; 2 Jiangsu Provincial Key Laboratory of Chronic Digestive Diseases, The First Affiliated Hospital with Nanjing Medical University, Nanjing, Jiangsu, China.; 3 Department of Pathology, Nippon Veterinary and Life Science University, Tokyo, Japan.; 4 Department of Pathology, Graduate School of Medicine, Fujita Health University, Toyoake, Japan.; 5 Department of Gastrointestinal Surgery, Graduate School of Medicine, The University of Tokyo, Tokyo, Japan.; 6 Department of General Surgery, The Affiliated Wuxi People's Hospital of Nanjing Medical University, Wuxi People's Hospital, Wuxi Medical Center, Nanjing Medical University, Wuxi, Jiangsu, China.; 7 Jiangsu Key Lab of Cancer Biomarkers, Prevention and Treatment, Collaborative Innovation Center for Personalized Cancer Medicine, Nanjing Medical University, Nanjing, Jiangsu, China.

**Keywords:** tumor-associated macrophages, exosomes, GPX4, palmitoylation, immunotherapy

## Abstract

**Background:**

Gastric cancer (GC) remains a major global health challenge, characterized by poor outcomes driven by an immunosuppressive tumor microenvironment (TME). Tumor-associated macrophages (TAMs), particularly the M2 subtype, are central mediators of immune evasion and therapeutic resistance. While tumor-derived exosomes are key regulators of intercellular communication, the mechanisms by which they modulate TAM fate remain unclear.

**Methods:**

Proteomic profiling, molecular assays, and *in vivo* models were used to identify GC-derived exosomal cargos regulating macrophage polarization. The CaMK2A-ZDHHC3-GPX4 axis was dissected using phosphorylation, palmitoylation, and genetic perturbation analyses. The therapeutic implications were evaluated through macrophage-specific GPX4 ablation and anti-PD-1/PD-L1 blockade in murine GC models.

**Results:**

We identified exosomal CaMK2A as a critical determinant of TAM polarization. Internalized CaMK2A phosphorylates ZDHHC3 at Thr176, enhancing GPX4 S-palmitoylation at Cys10, preventing its lysosomal degradation, and stabilizing GPX4 protein. This cascade suppresses macrophage ferroptosis and promotes M2 polarization, fostering tumor proliferation and metastasis. Conversely, GPX4 deletion in macrophages restrains tumor growth and synergizes with PD-1/PD-L1 blockade to enhance antitumor immunity. Clinically, GPX4 is upregulated in GC, enriched in TAMs, and predicts poor prognosis.

**Conclusions:**

Our study reveals a previously unrecognized CaMK2A-ZDHHC3-GPX4 signaling axis that couples ferroptosis resistance to immunosuppressive TAM polarization. Targeting GPX4 or disrupting exosomal CaMK2A signaling may reprogram the TME and potentiate immune checkpoint therapy in GC.

## 1. Introduction

Gastric cancer (GC) ranks as the fifth most prevalent malignancy worldwide in both incidence and mortality, with particularly high occurrence rates in East Asia, including China 1, 2. Owing to its insidious onset and progressive development, the majority of patients are diagnosed at advanced stages, resulting in a poor 5-year survival rate 3. Beyond surgical resection, chemotherapy remains the cornerstone treatment modality for advanced GC, often complemented by immunotherapy, targeted therapy, and other multimodal therapeutic approaches 4. Nevertheless, non-surgical treatment options for advanced GC remain constrained by pronounced tumor heterogeneity and biological complexity 5. Recent breakthroughs in immunotherapy have provided renewed optimism, particularly through the use of immune checkpoint inhibitors such as pembrolizumab and nivolumab 6, 7. Despite these advancements, the therapeutic efficacy of immunotherapy remains suboptimal, as only a limited subset of patients exhibit favorable responses. This limitation is largely attributed to the intricate tumor microenvironment (TME), comprising a diverse array of immune cells, stromal cells, endothelial cells, and fibroblasts 8, 9. Among these, tumor-associated macrophages (TAMs) constitute a predominant immune cell population that profoundly influences TME composition and function 10, 11. Macrophages display remarkable phenotypic plasticity, allowing polarization into distinct functional subsets. Classically activated M1 macrophages exhibit anti-tumorigenic activity, whereas alternatively activated M2 macrophages contribute to immune suppression, angiogenesis, and tumor progression 12, 13. Consequently, elucidating the molecular mechanisms governing macrophage polarization is critical for advancing our understanding of the immune landscape in GC.

Exosomes, as critical mediators of intercellular communication, play an essential role in modulating the TME 14. Through the transfer of proteins, nucleic acids, and lipids to recipient cells, exosomes can profoundly alter immune cell function and promote the formation of an immunosuppressive milieu 15. Tumor-derived exosomes have been shown to drive macrophage polarization toward the M2 phenotype, thereby enhancing tumor proliferation, invasion, and metastasis 16-19. Nonetheless, the specific molecular constituents underlying this reprogramming process, particularly exosomal proteins, remain insufficiently elucidated.

Calcium/calmodulin-dependent protein kinase II alpha is a multifunctional serine/threonine kinase that regulates a wide range of cellular processes, including signal transduction and metabolism 20-22. Aberrant CaMK2A activity has been associated with tumor cell proliferation and therapy resistance in several cancers 23-25. Nevertheless, the role of exosomal CaMK2A in shaping the TME, and specifically its influence on macrophage function, has not been elucidated.

Emerging evidence indicates that antitumor immune responses are intricately regulated by diverse lipid-derived post-translational modifications (PTMs) 26, 27, among which S-palmitoylation has garnered particular attention 28, 29. S-palmitoylation is a reversible lipid-protein modification involving the covalent attachment of palmitoyl groups to cysteine residues of substrate proteins, thereby modulating their stability, subcellular localization, and functional activity 30-33. Dysregulated protein palmitoylation has been implicated in tumorigenesis, immune evasion, and the progression of multiple pathological conditions 34-39. This modification is catalyzed by a family of 23 mammalian palmitoyltransferases (DHHCs), characterized by the conserved Asp-His-His-Cys motif, and is dynamically reversed by acyl protein thioesterases such as APT1/2 and members of the alpha/beta hydrolase domain-containing (ABHD) protein family 40. Among these enzymes, ZDHHC3 has been identified as a key regulator of substrate palmitoylation and has been associated with tumor development and progression 41, 42. Yet, the mechanisms governing ZDHHC3 activity within immune cells and its potential role in macrophage polarization within the TME remain to be elucidated.

Glutathione peroxidase 4 (GPX4), a selenocysteine-containing enzyme, catalyzes the reduction of phospholipid hydroperoxides (PLOOH) to their corresponding alcohols, thereby exerting potent antioxidant effects and serving as a key regulator of ferroptosis 43, 44. Stabilization of GPX4 prevents ferroptosis and promotes cell survival 45. Recent studies have suggested that ferroptosis not only influences tumor cells but also regulates macrophage function within the TME 46-48. Although GPX4 has been reported to be palmitoylated by ZDHHC8 and ZDHHC20 49, 50, other mechanisms underlying its palmitoylation, particularly the modifications in macrophages, still need to be explored.

Taken together, although exosomes, CaMK2A, ZDHHC3, and GPX4 have each been independently implicated in cancer biology and immune regulation, their potential interplay in orchestrating macrophage polarization and ferroptosis during GC progression remains largely unexplored. In this study, we demonstrate that exosomal CaMK2A derived from GC cells is internalized by macrophages, where it phosphorylates ZDHHC3, a palmitoyl acyltransferase that mediates GPX4 palmitoylation. This phosphorylation-dependent palmitoylation enhances GPX4 stability, reduces its lysosomal degradation, and ultimately facilitates M2 macrophage polarization while suppressing ferroptosis. These findings reveal a previously unrecognized regulatory axis that links exosomal CaMK2A to macrophage polarization and ferroptosis, providing new mechanistic insight into GC progression and identifying potential therapeutic targets for immunomodulation.

## 2. Materials and Methods

### 2.1 Patients and tissue samples

A total of 62 pairs of GC tissues and corresponding adjacent normal tissues were procured from patients at the First Affiliated Hospital with Nanjing Medical University between 2019 and 2020. The freshly resected specimens were promptly frozen in liquid nitrogen and subsequently stored at -80℃ until required for analysis. The clinical diagnoses of all patients were meticulously confirmed through expert pathological evaluation. This study was granted ethical approval by the Medical Ethics Committee of the First Affiliated Hospital with Nanjing Medical University (2024-SRFA-459), and written informed consent was obtained from all participants. Moreover, comprehensive clinical records and pathological data were systematically compiled for analysis.

### 2.2 Cell culture

The human gastric epithelial cell line GES-1, GC cell lines (HGC27 and AGS) and human leukemia monocytic cell line THP-1 were purchased from the Cell Bank of Type Culture Collection of the Chinese Academy of Sciences. The murine GC cell line (YTN16) was kindly provided by Dr. Masami Yamamoto, Dr. Tetsuya Tsukamoto and Dr. Sachiyo Nomura, and the cell line was cultured as described previously 51. Cells were maintained according to culture instructions and used within 6 months after initial culture.

### 2.3 Transfection and plasmid construction

ShRNA targeting CaMK2A and GPX4 was obtained from Sigma-Aldrich (USA). ShRNA was packaged in lentivirus vectors. CaMK2A, ZDHHC3 and GPX4 were cloned into pcDNA3.1 vector (GenePharma, China). Transfection of plasmid and shRNA was performed with Lipofectamine 3000 (Thermo Fisher, USA).

### 2.4 Exosome isolation and purification

Exosomes were isolated and purified from 1 × 10^7^ HGC27, AGS and GES-1 cells cultured in medium supplemented with 10% sEV-depleted fetal bovine serum (FBS). After 72 h of incubation, the cell culture supernatant was harvested and subjected to differential centrifugation to isolate sEVs. Specifically, the supernatant was first centrifuged at 300 × g for 10 min, followed by 2000 × g for 10 min, and then 10,000 × g for 30 min to eliminate cells, dead cells, and cellular debris. The resulting pellet was resuspended in phosphate-buffered saline (PBS) and subjected to ultracentrifugation at 100,000 × g for 70 min. The process was repeated after resuspension to ensure optimal sEV recovery. All centrifugation steps were carried out using a Beckman-optimized X100 ultracentrifuge.

For plasma-derived sEVs, isolation was performed using the sEVs Extraction and Purification Kit (Umibio, China) according to the manufacturer's instructions, starting with 1 mL of plasma. The procedure consisted of three stages: (1) removal of non-vesicular proteins, (2) extraction of sEVs, and (3) purification of sEVs. The protein concentration of the purified sEVs was determined using the Bicinchoninic Acid (BCA) Protein Assay Kit (ThermoFisher Scientific, USA).

### 2.5 Transmission electron microscopy (TEM)

For transmission electron microscopy analysis, exosome samples were first suspended in 2.5% glutaraldehyde and incubated for 2 h, followed by rinsing with PBS. Subsequently, 20 μL of the exosome suspension was applied to a carbon-coated copper grid (Electron Microscopy Sciences, USA), and any excess suspension was gently removed using filter paper. The grid was then stained with 3% phosphotungstic acid for 1 min to enhance the contrast of the exosomes. After drying, the morphology of the stained exosomes was examined under a Tecnai G2 F20 TEM (FEI Company, USA).

### 2.6 Exosome labeling and tracking

Exosome labeling was performed using Vybrant™ DiO Cell-Labeling Solution (Invitrogen, USA). A total of 1 mL of exosome suspension was supplemented with 5 μL of 3,3′-dioctadecyloxacarbocyanine perchlorate (DiO; green) solution and incubated at 37°C for 20 min. Following incubation, the suspension underwent three rounds of centrifugation, washing, and a 10-min recovery period. DiO-labeled exosomes were subsequently added to the conditioned medium of recipient cells. To assess the uptake of exosomal protein by recipient cells, cells containing FITC-labeled protein were co-cultured with the exosomes. At designated time points (0, 1, 2, 4, 6, 12, 24, or 48 h post-exosome treatment or co-culture), cells were fixed with 4% paraformaldehyde (Sigma-Aldrich) for 5 min and permeabilized using 0.01% Triton X-100 (Invitrogen, USA) for 10 min. To visualize the cytoskeleton, cells were stained with phalloidin conjugated to tetramethylrhodamine isothiocyanate (TRITC; red; R&D Systems) at a concentration of 150 nM for 20 min. The final images were captured using the Thunder Imager fast high-resolution inverted fluorescence imaging system (LEICA, Germany).

### 2.7 RNA isolation and Quantitative real-time polymerase chain reaction (qRT-PCR)

Total RNA was extracted from cells using the Trizol Kit (Invitrogen, USA). To this end, 1 mL of Trizol reagent was added to the cell and tissue lysate, and the mixture was incubated at room temperature for 10 min. After incubation, the mixture was centrifuged to isolate the supernatant, to which 200 µL of chloroform was added. Following centrifugation, the upper aqueous phase was carefully separated. The RNA was then precipitated by adding 200 µL of isopropanol to the supernatant, followed by another centrifugation step to collect the pellet. The pellet was washed with 75% ethanol and subsequently air-dried. Finally, the RNA pellet was dissolved in DEPC-treated water.

Reverse transcription was performed using the Takara RT reagent (Takara Bio, Japan). qRT-PCR was carried out on an ABI PRISM 7300 Sequence Detection System (Applied Biosystems, USA) using SYBR Premix Ex Taq™ II (Takara, China), following the manufacturer's protocol. Relative gene expression levels were calculated using the 2^-ΔΔCt^ method. The sequences of the primers used in this study are provided in **Supplementary Table 1**.

### 2.8 Western blot and immunoprecipitation (IP)

Total protein was extracted using RIPA buffer (Beyotime, China). The protein samples were then resolved on SDS-PAGE gels of appropriate concentration and subsequently transferred to a nitrocellulose membrane. Following a 1 h blocking step with 5% BSA, the membrane was incubated with specific primary antibodies overnight at 4℃. Afterward, the membrane was incubated with the corresponding secondary antibodies for 2 h at room temperature. Protein detection was carried out using ECL reagent (Millipore, USA). A list of the antibodies used is provided in **Supplementary Table 2**.

For IP, proteins extracted from cell lysates were incubated with protein A/G-agarose beads (Santa Cruz Biotechnology, USA) and the corresponding antibody for 4 h at 4 ℃. The mixture was then centrifuged, and the supernatant was discarded. After washing the beads three times, the beads were incubated with the protein lysate overnight at 4 ℃. Following centrifugation, the supernatant was discarded, and the immunoprecipitate was washed thoroughly. To elute the proteins, the beads were boiled for 5 min at 95℃ in the presence of SDS. The immunoprecipitated proteins were analyzed by Western blotting and further characterized by mass spectrometry (Thermo Scientific, USA).

### 2.9 Flow cytometry

To assess CD206^+^ macrophages, PMA-stimulated THP-1 cells were first harvested and fixed overnight in 1% paraformaldehyde at 4 °C. Following fixation, cells were resuspended in flow cytometry buffer (1 × PBS supplemented with 1% FBS) and incubated with anti-CD206 antibody for 30 min at room temperature. To evaluate reactive oxygen species (ROS) levels, cells were stained with BODIPY 581/591 C11, which specifically detects ROS, and analyzed using flow cytometry (Beckman Coulter, USA).

### 2.10 Immunohistochemistry (IHC)

Tissues were fixed in 10% formalin for 24 h and subsequently embedded in paraffin. The embedded tissues were sectioned to the desired thickness and incubated with specific primary antibodies overnight at 4 °C. After two rinses, the sections were incubated with species-specific secondary antibodies for 1 h at 37 °C. Following incubation, the sections were stained with 3,3-diaminobenzidine (DAB) solution, counterstained with hematoxylin, and then examined under a microscope (Nikon, Japan). The antibodies used were listed in **Supplementary Table 2**.

### 2.11 Immunofluorescence (IF)

Cells were seeded in 35-mm culture plates and incubated overnight. Following fixation with 4% paraformaldehyde and permeabilization with 0.1% Triton X-100, the cells were incubated with primary antibodies overnight at 4 °C. After rinsing, the cells were incubated with species-specific secondary antibodies for 2 h at room temperature in the dark. Nuclei were stained with DAPI, and fluorescence images were acquired using a Leica SP5 confocal microscope system (Leica Microsystems, Germany). The antibodies used were listed in **Supplementary Table 2**.

### 2.12 Colony-forming assay

Cells were seeded in 6-well plates at a density of 500 cells per well and cultured for two weeks to allow colony formation. After incubation, colonies were fixed with 4% paraformaldehyde for 10 min and subsequently stained with crystal violet solution (Kaigen, China) for 30 min. The stained colonies were then imaged and quantified.

### 2.13 5-Ethynyl-2′-deoxyuridine (EdU) assay

To assess DNA synthesis, GC cells were seeded in 96-well plates at a density of 10,000 cells per well. After a 2 h incubation with EdU solution (Beyotime, China), cells were fixed with 4% paraformaldehyde. Following fixation, the reaction mixture was applied according to the manufacturer's protocol, and nuclei were counterstained with Hoechst 33342. Fluorescence images were captured using a Nikon fluorescence microscope (Nikon, Japan).

### 2.14 Transwell assay

Transwell inserts (Millipore, USA) were positioned in 24-well plates, with the lower chamber containing 600 μL of RPMI 1640 supplemented with 10% FBS. Meanwhile, 20,000 cells in 200 μL of RPMI 1640 were seeded into the upper chambers. After 48 h, the cells were fixed with methanol, stained with crystal violet, and subsequently imaged using an inverted microscope (Nikon, Japan).

### 2.15 GST pull-down assay

Purified Flag-GPX4 was incubated with glutathione Sepharose 4B (Catia, China) bound to GST or GST-CaMK2A in the binding buffer at 4 °C overnight. After washing three times, the pull-down samples were analyzed by Western blot.

### 2.16 Ubiquitination assay

Ubiquitination of GPX4 was assessed using denaturing IP (d-IP) and TUBE pull-down assays. For d-IP, cells were lysed in SDS-denaturing buffer (62.5 mM Tris-HCl, pH 6.8, 10% glycerol, 2% SDS, 1.5% β-mercaptoethanol) and incubated at 95 °C for 10 min. The resulting cell lysates were then diluted tenfold in native lysis buffer (50 mM Tris-HCl, pH 7.4, 0.5% Triton X-100, 200 mM NaCl, 10% glycerol). Following centrifugation, the supernatants were incubated with the specified antibodies overnight at 4 °C for IP. The immunoprecipitates were washed three times and analyzed by Western blot.

Endogenous ubiquitination of GPX4 was further evaluated in the immunoprecipitated lysates using TUBE-Flag, following the manufacturer's protocol for the pull-down assay.

### 2.17 Labeling, click reaction, and streptavidin pulldown

Cells labeled with alkynyl probes were lysed using lysis buffer (50 mM TEA-HCl, pH 7.4, 150 mM NaCl, 0.1% Triton X-100, 0.2% SDS, and protease inhibitors), followed by a click reaction with biotin picolyl azide. The proteins were precipitated by adding nine volumes of 100% methanol and incubated for 2 h at -80 °C. The precipitates were then collected by centrifugation at 14,000 × g for 10 min.

The resulting pellets were resuspended in 100 μL of suspension buffer (50 mM Tris-HCl, pH 7.4, 150 mM NaCl, 5 mM EDTA, and 2% SDS), and subsequently diluted tenfold with immunoprecipitation buffer (50 mM Tris-HCl, pH 7.4, 150 mM NaCl, 5 mM EDTA, and 0.5% NP40). Samples were incubated with streptavidin agarose beads at room temperature for 2 h. After incubation, the protein-bound streptavidin agarose beads were washed three times with immunoprecipitation buffer, and the bound proteins were eluted using elution buffer (10 mM EDTA, pH 8.2, and 95% formamide) for 10 min at 95 °C. The eluted samples were subsequently analyzed by Western blot.

### 2.18 Acyl-PEG exchange (APE) assay

Cells were lysed using lysis buffer (50 mM TEA-HCl, pH 7.4, 150 mM NaCl, 1% Triton X-100, 2% SDS) supplemented with protease and phosphatase inhibitors. The lysates were incubated with 10 mM TCEP for 30 min, followed by alkylation with 25 mM NEM for 2 h at room temperature. The protein mixture was subjected to methanol-chloroform precipitation, and subsequently treated with NH_2_OH to cleave palmitoylation thioester bonds at 37 °C for 1 h.

After the reaction, proteins were again precipitated by methanol-chloroform and incubated with 2 mM PEG at room temperature for 1 h. Following this, proteins were precipitated once more using methanol-chloroform, resuspended in loading buffer, and boiled at 95 °C for 3 min. The samples were then analyzed by Western blot.

### 2.19 Proximity Ligation Assay (PLA)

To detect the interaction between CaMK2A and ZDHHC3, cells were seeded on sterile coverslips in 12-well plates and cultured under standard conditions. After the indicated treatments, cells were fixed with 4% paraformaldehyde for 15 min, permeabilized, and blocked according to the manufacturer's instructions. The cells were then incubated overnight at 4 °C with mouse anti-Flag antibody and rabbit anti-ZDHHC3 antibody. After washing, cells were incubated with anti-mouse MINUS and anti-rabbit PLUS PLA probes, followed by ligation and amplification using the Duolink *In situ* PLA kit (Sigma-Aldrich) according to the manufacturer's protocol. Nuclei were counterstained with DAPI, and PLA signals were visualized using a Zeiss LSM 880 confocal microscope.

### 2.20 Isolation of Bone Marrow-Derived Macrophages (BMDMs)

Mice were euthanized by cervical dislocation under isoflurane anesthesia. After sterilization in 75% ethanol for 5 min, femurs and tibias were aseptically isolated. Bone marrow cells were flushed from the bones using serum-free RPMI-1640 medium. The collected cell suspension was centrifuged at 1500 rpm for 5 min, and the cell pellet was resuspended in red blood cell lysis buffer for 5 min to remove erythrocytes. After an additional centrifugation step at 1500 rpm for 5 min, the cells were cultured in RPMI-1640 supplemented with 10% FBS and 20 ng/mL M-CSF for 7 days to generate BMDMs for subsequent experiments.

### 2.21 Animal models

For the subcutaneous tumor mouse model, 5-week-old female BALB/C nude mice were randomly assigned to different experimental groups. A total of 2 × 10^6^ HGC27 cells and 2 × 10^6^ PMA-induced THP-1 cells (treated with or without GC-derived exosomes) were resuspended in 200 μL PBS and injected as a mixture into the groin area of the mice. After 3 weeks, the mice were sacrificed, and tumor volume and size were measured. Tumor volume was calculated using the formula: V = length × width² × 0.5.

For the liver metastasis tumor model, 5-week-old female BALB/C nude mice were randomly assigned to different groups. A mixture of 2 × 10^6^ luciferase-labeled HGC27 cells and 2 × 10^6^ PMA-induced THP-1 cells (treated with or without GC-derived exosomes) was resuspended in 200 μL PBS and injected into the spleen of the mice. After 3 weeks, *in vivo* imaging was performed using a Calliper Life Sciences imaging system (Hopkinton, MA, USA). Mice were then sacrificed, and their livers were harvested for imaging and H&E staining.

The Gpx4 loxP mouse in C57BL/6J background was generated by flanking exon 2 to exon 4 of Gpx4 with loxP sites from M.Q. MICROBE Co., Ltd (China). The heterozygous Gpx4 loxP mice were intercrossed to obtain homozygous Gpx4 loxP mice. Homozygous Gpx4 knockout mice were crossed with LysM promoter-driven Cre recombinase mice (LysM-Cre mice) to generate Gpx4-macrophage KO mice for inducible macrophage-specific deletion of Gpx4 of C57BL/6J background. YTN16 cells (5×10^6^/tumor) in 100 μL PBS/Matrigel (1:1, v/v) were subcutaneously injected into the the dorsal flank of 4-week-old C57BL/6J mice to generate syngeneic tumors. At the end point, the mice were sacrificed, and tumor volume and size were measured. Tumor volume was calculated using the formula: V = length × width² × 0.5. The primers for genotyping were listed in **Supplementary Table 1**.

All animal experimental procedures were approved by the Animal Ethics Committee of Nanjing Medical University (IACUC-2504059). BALB/C nude mice were purchased from the Laboratory Animal Centre of Nanjing Medical University and were housed under pathogen-free conditions.

### 2.22 Statistical analysis

Statistical significance between two groups was assessed using the Student's t-test. Pearson correlation analysis was performed to determine the corresponding R and *p* values for the scatter plots. One-way analysis of variance (ANOVA), followed by Tukey's multiple comparisons test, was used to evaluate differences among multiple groups. Data are presented as mean ± standard deviation (SD). A *p*-value of less than 0.05 was considered statistically significant. Statistical significance is indicated in the figures and figure legends as follows: no significance [ns], *p < 0.05, **p < 0.01 and ***p < 0.001. All experiments were independently repeated a minimum of three times.

## 3. Results

### 3.1 GC cells induce M2 polarization of macrophages in an exosome-dependent manner

**Figure 1:**
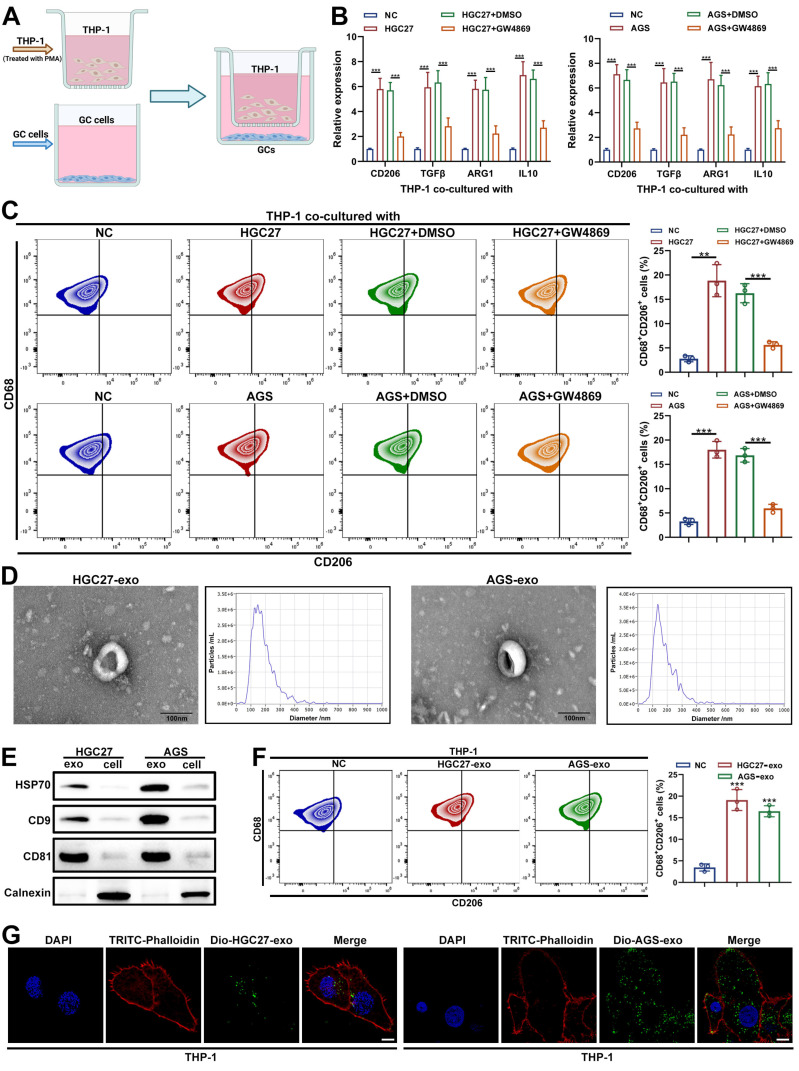
** GC cells induce M2 polarization of macrophages in an exosome-dependent manner. A.** Schematic illustration of the co-culturing system for macrophages and GC cells using a transwell chamber (created in https://BioRender.com). **B.** qRT-PCR was utilized to detect the levels of M2 markers (CD206, TGFβ, ARG1 and IL10) in THP-1 cells with or without the co-culture with GC cells co-culture and the treatment of GW4869 (5μM) on GC cells. **C.** Flow cytometry was applied to detect macrophage marker (CD68) and M2 macrophage-related phenotypic marker (CD206) on the surfaces of macrophages in each group. The ratio of CD206-positive plus CD68-positive (CD68^+^CD206^+^) macrophages of each group were quantitated. **D.** TEM images and NTA analysis results of HGC27-exo or AGS-exo. Scale bar: 100 nm. **E.** Western blot of exosome markers in extracted exosomes from GC cells. **F.** Flow cytometry was applied to measure CD68^+^CD206^+^ macrophages in each group of THP-1 cells with or without GC cells-derived exosome treatment. **G.** Fluorescence microscopy images illustrated the process of GC cells-derived exosomes transmitted to macrophages. GC cells-derived exosomes were labeled by DiO (green). TRITC-Phalloidin (red) was adopted for labeling cytoskeleton of THP-1 cells. Scale bar: 10 μm. Data are presented as mean ± SD of at least three independent experiments. *p < 0.05, **p < 0.01, ***p < 0.001. n.s., no significance.

To examine the impact of GC cells on macrophage polarization, HGC27 or AGS cells were cocultured with PMA-induced THP-1 cells in a Transwell system (**Figure 1A**). PMA-induced THP-1 cells cultured alone served as negative controls. After 48 h, qRT-PCR analysis revealed markedly increased expression of M2-associated markers (ARG1, CD206, TGFβ, and IL10) in THP-1 cells cocultured with GC cells, whereas M1 markers (TNFα, NOS2, CXCL9, and CXCL10) remained unchanged. Treatment with the exosome release inhibitor GW4869 abrogated the upregulation of M2 markers but had no effects on M1 markers (**Figure 1B and Supplementary Figure S1A-B**). Flow cytometric analysis confirmed an elevated proportion of CD68⁺CD206⁺ macrophages following coculture, which was also diminished by GW4869 (**Figure 1C**). These findings indicate that GC cells promote M2 polarization of macrophages through an exosome-dependent mechanism.

To validate this, exosomes were isolated from HGC27 and AGS cells. TEM revealed typical bilayer, saucer-shaped vesicles (50-200 nm), and nanoparticle tracking analysis confirmed a median diameter of approximately 100 nm (**Figure 1D**). Western blot showed enrichment of exosomal markers (HSP70, CD9, CD81) and minimal calnexin expression, confirming purity (**Figure 1E**). Functionally, GC-derived exosomes increased the expression of M2-associated markers and CD68⁺CD206⁺ macrophage population, whereas M1 markers (TNFα, NOS2, CXCL9, and CXCL10) remained unchanged (**Figure 1F and Supplementary Figure S1C-D**). Moreover, DiO-labeled GC exosomes were readily internalized by THP-1 cells within 24 h (**Figure 1G**). Taken together, these data demonstrate that GC-derived exosomes mediate macrophage M2 polarization *in vitro*.

### 3.2 GC-derived exosomal CaMK2A promotes M2 polarization of macrophages

**Figure 2:**
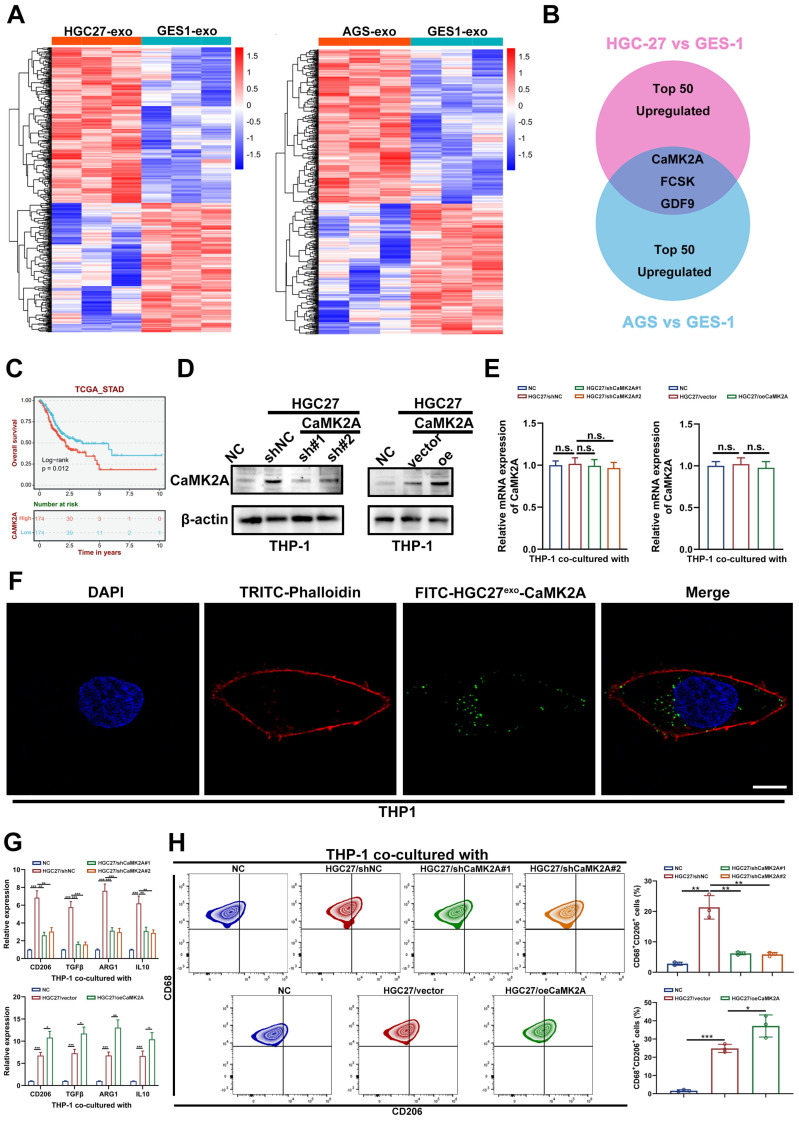
** GC-derived exosomal CaMK2A promotes M2 polarization of macrophages. A.** Proteomic profiling of exosomes isolated from GC cells (HGC27 and AGS cells) and normal gastric epithelial cells (GES-1 cells). **B.** The intersection of the top 50 differential upregulated proteins from both groups. **C.** Survival analysis of CaMK2A in TCGA-STAD cohort. **D.** Western blot was adopted to detect the expression levels of CaMK2A in THP-1 cells co-cultured with HGC27 cells transfected with shCaMK2A, oeCaMK2A, or untransfected. **E.** qRT-PCR was applied to evaluate the expression levels of CaMK2A in THP-1 cells co-cultured with GC cells, either transfected with shCaMK2A, oeCaMK2A, or untransfected. **F.** Fluorescence microscopy images illustrated the process of HGC27 cells-derived exosomal CaMK2A transmitted to macrophages. HGC27 cells-derived CaMK2A were labeled by FITC (green). TRITC-Phalloidin (red) was adopted for labeling cytoskeleton of THP-1 cells. Scale bar: 10 μm. **G.** qRT-PCR was adopted to measure the levels of M2 markers in THP-1 cells co-cultured with HGC27 cells transfected with shCaMK2A, oeCaMK2A, or untransfected. **H.** Flow cytometry was applied to measure CD68^+^CD206^+^ macrophages in THP-1 cells co-cultured with HGC27 cells, either transfected with shCaMK2A, oeCaMK2A, or untransfected. Data are presented as mean ± SD of at least three independent experiments. *p < 0.05, **p < 0.01, ***p < 0.001. n.s., no significance.

To elucidate the molecular mechanisms underlying GC-derived exosome-induced macrophage polarization, we conducted a quantitative proteomic analysis of exosomes isolated from GC cells (HGC27 and AGS) and normal gastric epithelial cells (GES-1) (**Figure 2A**). Comparative protein profiling identified three consistently upregulated proteins, CaMK2A, FCSK, and GDF9, shared between both GC-derived exosome datasets (**Figure 2B**). Among these, Kaplan-Meier survival analysis (https://rookieutopia.hiplot.com.cn/) revealed that only CaMK2A expression correlated significantly with poor prognosis in patients with GC (**Figure 2C and Supplementary Figure S2A**). Examination of exosomes from paired GC and adjacent normal tissues further confirmed that CaMK2A was markedly enriched in GC-derived exosomes (**Supplementary Figure S2B**), prompting us to investigate its functional relevance in GC-macrophage communication.

Western blot analysis demonstrated that coculture of GC cells with THP-1 macrophages resulted in a robust increase in CaMK2A protein levels within THP-1 cells. This elevation was abrogated by CaMK2A knockdown in GC cells and further amplified upon CaMK2A overexpression (**Figure 2D**). In contrast, qRT-PCR revealed no significant alterations in CaMK2A mRNA, suggesting protein transfer rather than transcriptional induction (**Figure 2E**). Consistently, fluorescence microscopy showed that FITC-labeled CaMK2A from GC cells was internalized by THP-1 macrophages after 24 h of coculture, confirming exosomal transfer of CaMK2A (**Figure 2F and Supplementary Figure S2C**).

Functionally, coculture with CaMK2A-overexpressing GC cells significantly elevated the expression of M2-associated markers in THP-1 macrophages, whereas CaMK2A silencing reversed this phenotype (**Figure 2G and Supplementary Figure S2D-E**). M1 marker expression remained unchanged (**Supplementary Figure S2F-I**). Flow cytometric analysis further validated that the proportion of CD68⁺CD206⁺ macrophages was increased by exosomal CaMK2A and diminished by its knockdown (**Figure 2H and Supplementary Figure S2J-K**). Collectively, these findings demonstrate that GC-derived exosomal CaMK2A is actively transferred to macrophages, driving M2 polarization and promoting an immunosuppressive phenotype.

### 3.3 Exosomal CaMK2A treated macrophages in turn enhance malignant behaviors of GC cells

**Figure 3:**
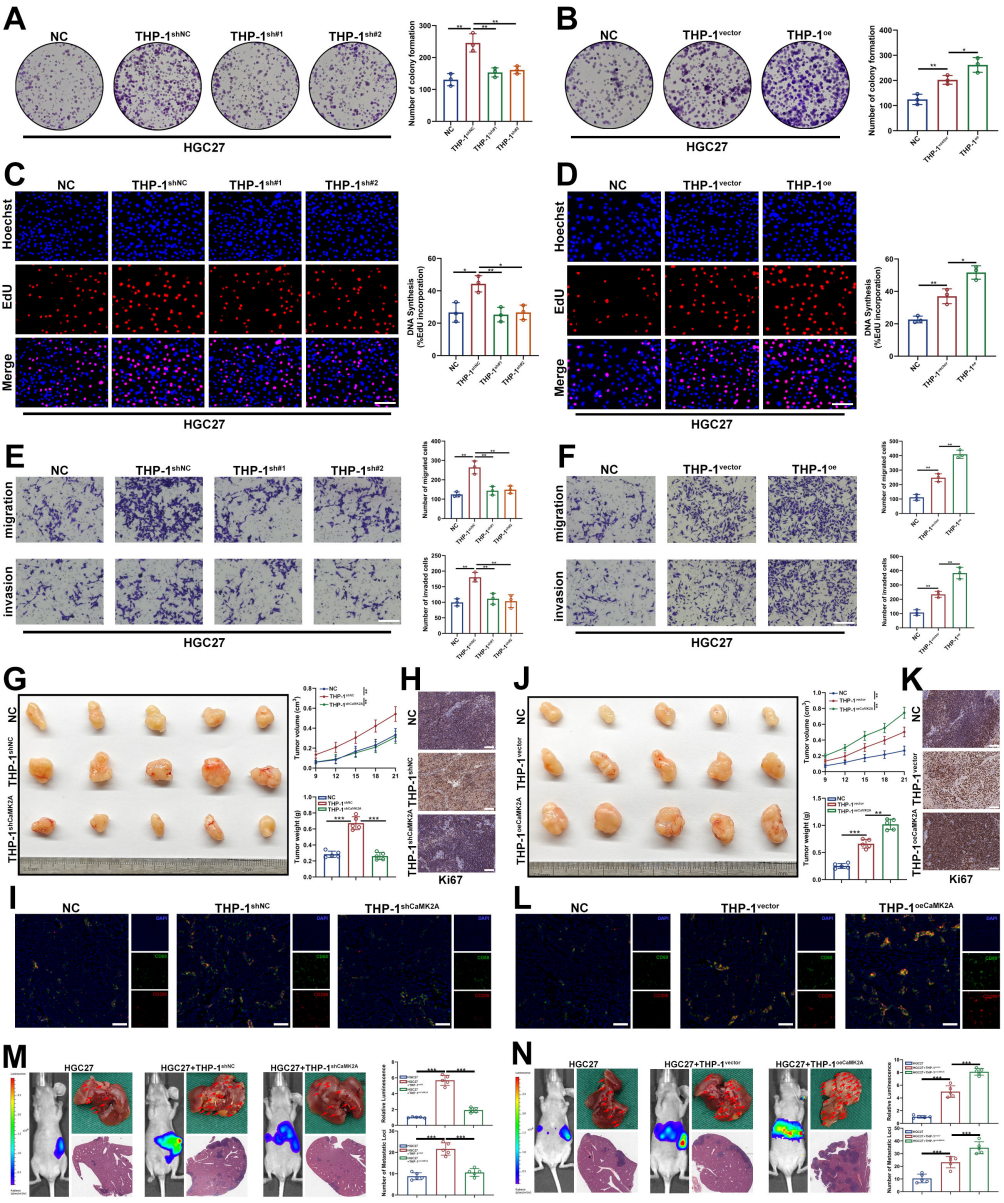
** Exosomal CaMK2A treated macrophages in turn enhances malignant behaviors of GC cells. A-B.** Colony formation assay was used to determine the effects of THP-1 cells on the proliferation of GC cells. THP-1 cells were pre-cocultured with HGC27 cells transfected with CaMK2A overexpressing or silencing. **C-D.** EdU assay was utilized to explore the influence of pre-treated THP-1 cells on the proliferation of HGC27 cells. Scale bar: 100 μm. **E-F.** Transwell assay was performed to detect the effects of pre-treated THP-1 cells on the migration and invasion of HGC27 cells. **G, J.** Gross images of tissue specimens from the subcutaneous xenograft tumor model in nude mice. Tumor growth rate and tumor weight of the model were also shown. Scale bar: 100 μm. **H, K.** IHC staining of Ki-67 of indicated groups from the subcutaneous xenograft tumor model in nude mice. Scale bar: 50 μm. **I-L.** mIHC analysis of CD68 and CD206 of indicated groups from the subcutaneous xenograft tumor model in nude mice. Scale bar: 100 μm. **M-N.** *In vivo* bioluminescent imaging assay, gross images and H&E staining for hepatic metastases for mice in each group. Data are presented as mean ± SD of at least three independent experiments. *p < 0.05, **p < 0.01, ***p < 0.001. n.s., no significance.

To determine the functional consequences of exosomal CaMK2A-induced macrophage polarization on tumor behavior, THP-1 macrophages pre-cocultured with GC cells transfected with CaMK2A-overexpressing or CaMK2A-silenced constructs were subsequently incubated with parental GC cells. Colony formation and EdU incorporation assays demonstrated that macrophages preconditioned by GC cells significantly enhanced GC cell proliferation, whereas this effect was abrogated when macrophages were educated by CaMK2A-knockdown GC cells. Conversely, CaMK2A overexpression in GC cells further augmented macrophage-mediated tumor cell proliferation (**Figure 3A-D and Supplementary Figure S3A-D**). Consistent results were observed in Transwell migration and invasion assays. Coculture with GC-educated macrophages markedly promoted the migratory and invasive capacities of GC cells, effects that were abolished upon CaMK2A silencing and further potentiated by CaMK2A overexpression (**Figure 3E-F and Supplementary Figure S3E-F**).

To further determine whether the tumor-promoting phenotype of GC-educated macrophages was mediated by exosomes, we isolated exosomes from control, CaMK2A-knockdown, or CaMK2A-overexpressing GC cells and directly used them to treat THP-1 macrophages before coculture with parental GC cells. Colony formation and EdU assays showed that exosome-treated THP-1 macrophages significantly promoted the proliferation of HGC27 and AGS cells. This effect was attenuated when macrophages were treated with exosomes from CaMK2A-silenced GC cells, but was further enhanced by exosomes from CaMK2A-overexpressing GC cells (**Supplementary Figure 3G-J**). Consistently, Transwell assays demonstrated that exosome-educated THP-1 macrophages markedly increased the migratory and invasive capacities of GC cells. These effects were weakened by exosomes from CaMK2A-knockdown GC cells and further strengthened by exosomes from CaMK2A-overexpressing GC cells (**Supplementary Figure 3K-L**). These findings indicate that exosomal CaMK2A reprograms macrophages into a tumor-promoting phenotype that facilitates GC cell aggressiveness.

To validate these observations *in vivo*, xenograft and hepatic metastasis models were established in nude mice. Subcutaneous coinjection of HGC27 and THP-1 cells that had been cocultured with GC cells significantly increased tumor volume and weight, accompanied by elevated Ki-67 expression and enrichment of CD68⁺CD206⁺ macrophages in tumor tissues as revealed by mIHC. These effects were markedly attenuated when macrophages were preconditioned with CaMK2A-knockdown GC cells, but further amplified when educated by CaMK2A-overexpressing GC cells (**Figure 3G-L**). Similarly, in the hepatic metastasis model, macrophages educated by GC cells promoted metastatic burden, which was diminished by CaMK2A silencing and enhanced by its overexpression (**Figure 3M-N**). To conclude, these results demonstrate that GC-derived exosomal CaMK2A-activated macrophages potentiate GC progression and metastasis by promoting tumor cell proliferation, migration, and invasion, thereby reinforcing a tumor-supportive immune microenvironment.

### 3.4 Exosomal CaMK2A upregulates GPX4, thus inhibiting ferroptosis and M2 polarization in macrophages

**Figure 4:**
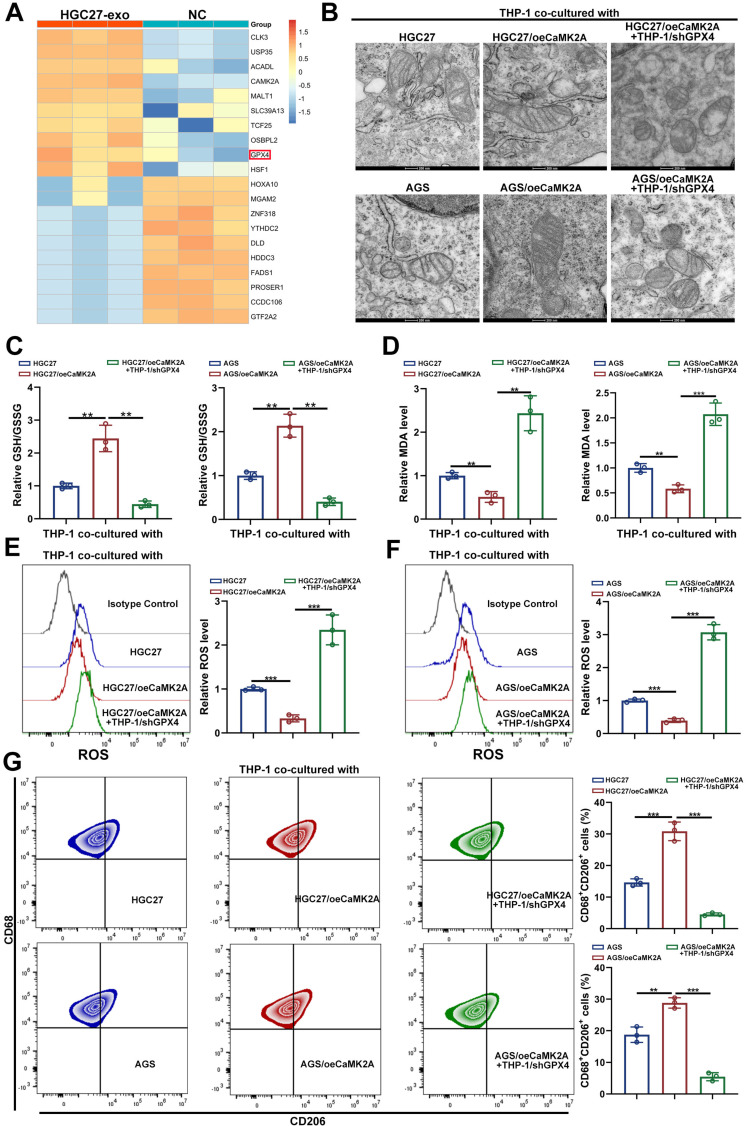
** Exosomal CaMK2A upregulates GPX4, thus inhibiting ferroptosis and M2 polarization in macrophages. A.** Proteomic profiling of THP-1 cells incubated with exosomes derived from either HGC27 cells or control cells. **B.** Representative TEM images of mitochondria in THP-1 cells of each group. **C, D.** Levels of GSH/GSSG ratio and MDA in THP-1 cells of each group. **E, F.** Relative lipid ROS levels in THP-1 cells transfected as indicated determined by BODIPY C11 staining following FACS analysis. **G.** Flow cytometry was applied to measure CD68^+^CD206^+^ macrophages in THP-1 cells of each group. Data are presented as mean ± SD of at least three independent experiments. *p < 0.05, **p < 0.01, ***p < 0.001. n.s., no significance.

To further elucidate the molecular mechanisms by which exosomal CaMK2A regulates macrophage function, we performed proteomic profiling of THP-1 cells incubated with or without exosomes derived from HGC27 cells. Differential expression analysis revealed a significant enrichment of proteins associated with the ferroptosis pathway, with multiple ferroptosis-suppressive proteins upregulated following exosome treatment (**Supplementary Figure S4A-B**). Among these, GPX4, a key ferroptosis inhibitor, was prominently elevated (**Figure 4A and Supplementary Figure S4C**).

TEM showed that coculture with HGC27 or AGS cells overexpressing CaMK2A markedly preserved mitochondrial integrity in THP-1 macrophages, indicative of ferroptosis suppression, whereas this protection was lost upon GPX4 knockdown (**Figure 4B**). Consistent findings were observed with ferroptosis-related biochemical indicators: levels of malondialdehyde (MDA) and the GSSG/GSH ratio were significantly reduced in the CaMK2A-overexpressing group, and reversed when GPX4 was silenced (**Figure 4C-D**).

Flow cytometry further demonstrated that GC cells overexpressing CaMK2A markedly reduced intracellular ROS accumulation in macrophages, whereas CaMK2A depletion restored ROS production (**Figure 4E-F**). Functionally, CaMK2A-overexpressing GC cells significantly increased the proportion of CD68⁺CD206⁺ macrophages, while GPX4 silencing abrogated this effect (**Figure 4G**). Consistent results were obtained at the transcriptional level, where M2-associated markers were downregulated upon GPX4 knockdown (**Supplementary Figure S4D-E**).

To further clarify the relationship between GPX4 deficiency, ferroptotic stress, and macrophage polarization, we performed rescue experiments using the ferroptosis inhibitor Ferrostatin-1 (Fer-1). Flow cytometric analysis showed that GPX4 silencing markedly reduced the proportion of CD68⁺CD206⁺ macrophages induced by coculture with HGC27^exo^ or AGS^exo^ cells, whereas Fer-1 treatment partially restored this population (**Supplementary Figure S4F**). Consistently, qRT-PCR analysis demonstrated that the expression of M2-associated markers was significantly decreased upon GPX4 knockdown, but was also partially rescued by Fer-1 treatment (**Supplementary Figure S4G**). The results suggest that the reduction in M2-associated features caused by GPX4 deficiency is at least partly attributable to enhanced ferroptotic stress. At the same time, the incomplete rescue by Fer-1 indicates that ferroptosis and M2 polarization are not simply linked in a linear upstream-downstream manner. Rather, GPX4 appears to coordinately regulate ferroptosis-related phenotypes and M2-like polarization, and both processes may jointly contribute to macrophage functional reprogramming. In conclusion, these findings demonstrate that exosomal CaMK2A enhances GPX4 stability in macrophages, suppressing ferroptosis and promoting M2 polarization, thereby fostering an immunosuppressive and tumor-supportive microenvironment.

### 3.5 S-palmitoylation stabilizes GPX4 by inhibiting polyubiquitination and lysosomal degradation

**Figure 5:**
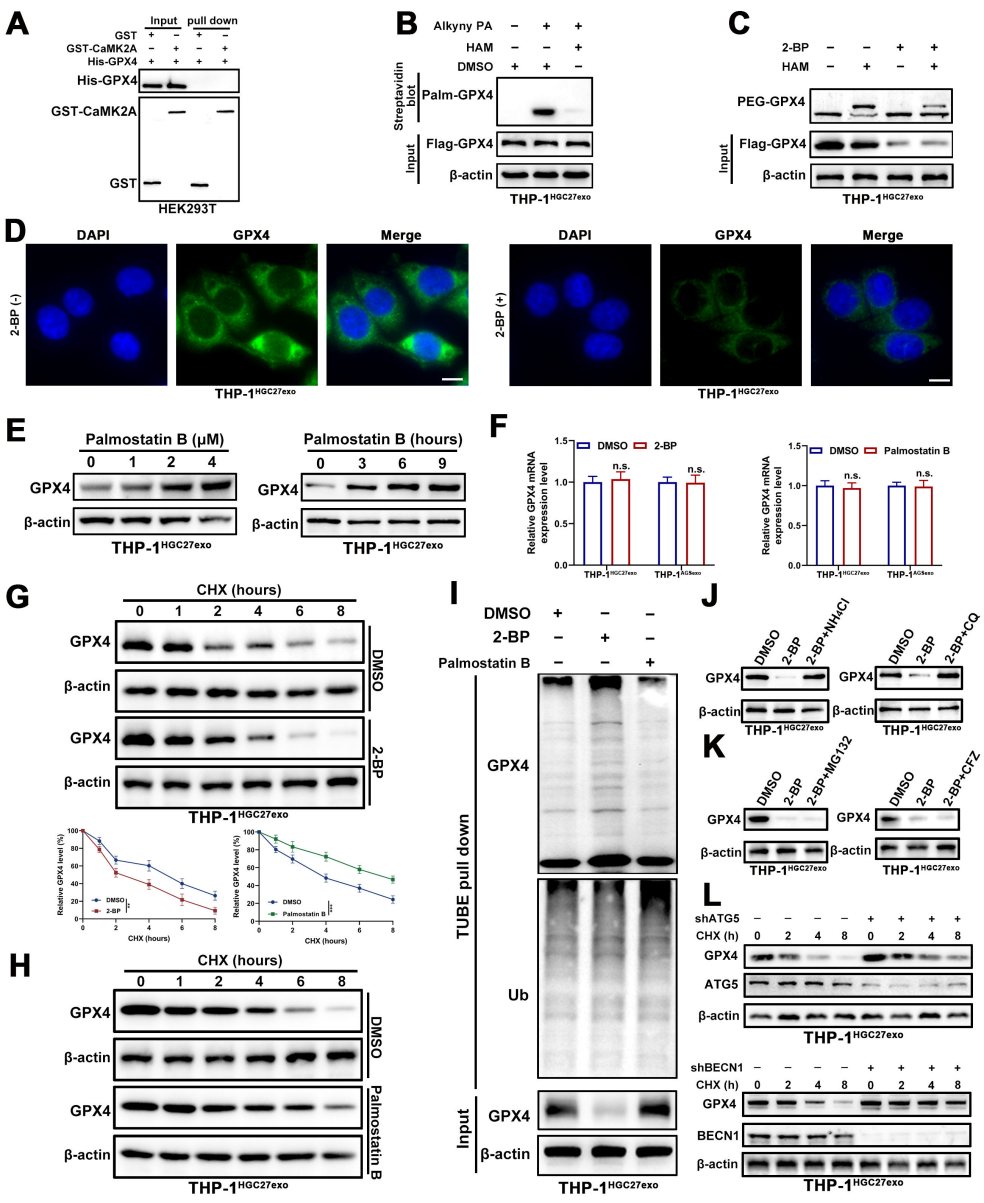
** S-palmitoylation stabilizes GPX4 by inhibiting polyubiquitination and lysosomal degradation. A.** GST-pull down was performed to determine the interaction between CaMK2A and GPX4. **B.** THP-1 cells transfected with Flag-GPX4 were metabolically labeled with 50 μM alkynyl palmitic acid (Alkyny PA) for 4 h by click reaction and subjected to streptavidin pulldown with or without the treatment of HAM, followed by Western blot using indicated antibodies. **C.** GPX4 palmitoylation in THP-1 cells were analyzed by the APE assays, upon 2-BP treatment (50 μM) in the absence or presence of HAM. PEG-GPX4 bands indicated palmitoylated GPX4. **D.** THP-1 cells were subjected to confocal analysis in the absence or presence of 2-BP. Scale bar: 10 μm. **E.** Western blot was used for detection of GPX4 protein levels in THP-1 cells treated as indicated amount and time of Palmostatin B. **F.** Detecting GPX4 mRNA levels in THP-1 cells treated with 50 μM 2-BP or 2 μM palmostatin B for 24 h and subsequently subjected to qRT-PCR. **G-H.** Detecting GPX4 protein levels in THP-1 cells treated with 2-BP (G) or palmostatin B (H) and subsequently subjected to the CHX chase assay for indicated times. **I.** Detecting GPX4 ubiquitination levels in THP-1 cells treated with palmostatin B and subsequently subjected to TUBE-pull down assay. **J-K.** Western blot analysis for GPX4 protein levels in THP-1 cells treated as indicated. Cells were treated with 2-BP (50 μM), MG132 (20 mM), Carfilzomib (100 nM), NH_4_Cl (20 mM), or CQ (100 mM) for 5 h before collection. **L.** Cells were transduced with control shRNA or shRNA targeting ATG5 or BECN1, followed by CHX chase analysis at the indicated time points. Western blot was performed to detect the degradation of GPX4. Data are presented as mean ± SD of at least three independent experiments. *p < 0.05, **p < 0.01, ***p < 0.001, n.s., no significance.

To elucidate the mechanism by which CaMK2A enhances GPX4 expression in macrophages, we first considered its kinase function. Given that CaMK2A is a serine/threonine kinase, we hypothesized that it might directly phosphorylate GPX4. However, GST pull-down assays showed no direct interaction between CaMK2A and GPX4, suggesting the involvement of intermediate regulators (**Figure 5A**). S-palmitoylation has emerged as an increasingly important PTM in recent years, and GPX4 has previously been reported to undergo palmitoylation. We therefore investigated whether GPX4 is palmitoylated in macrophages exposed to GC-derived exosomes. Using metabolic labeling with the palmitate analog Alk-C16, we confirmed that ectopically expressed Flag-GPX4 was robustly palmitoylated in THP-1 cells treated with GC-derived exosomes (THP-1^HGC27exo^ and THP-1^AGSexo^). This modification was largely abolished by hydroxylamine (HAM), confirming its palmitoylation specificity (**Figure 5B and Supplementary Figure S5A**). Endogenous GPX4 palmitoylation was further verified via APE assays. Treatment with 2-bromopalmitate (2-BP), a broad-spectrum palmitoylation inhibitor, markedly reduced both GPX4 palmitoylation and total protein levels (**Figure 5C and Supplementary Figure S5B**), findings corroborated by confocal microscopy (**Figure 5D and Supplementary Figure S5C**). Conversely, inhibition of depalmitoylating enzymes acyl-protein thioesterase 1/2 (APT1/2) with Palmostatin B increased GPX4 abundance (**Figure 5E and Supplementary Figure S5D**).

To directly assess the functional relevance of palmitoylation to GPX4 stability, cycloheximide (CHX) chase assays were conducted. Blocking palmitoylation with 2-BP shortened the GPX4 half-life, whereas Palmostatin B treatment prolonged it, without altering GPX4 mRNA expression (**Figure 5F-H** and **Supplementary Figure S5E-F**). Consistently, TUBE pull-down assays demonstrated increased polyubiquitination of GPX4 upon 2-BP treatment and reduced ubiquitination following Palmostatin B exposure (**Figure 5I and Supplementary Figure S5G**).

Furthermore, lysosomal inhibitors chloroquine (CQ) and NH₄Cl effectively rescued the 2-BP-induced reduction in GPX4 levels, whereas proteasome inhibitors (carfilzomib and MG132) did not (**Figure 5J-K and Supplementary Figure S5H-I**). To further strengthen the evidence that GPX4 degradation is mediated through the autophagy-lysosomal pathway rather than relying solely on pharmacological inhibition by CQ, we genetically disrupted key autophagy regulators (ATG5 and BECN1) in exosome-educated macrophages. CHX chase assays showed that knockdown of ATG5 markedly delayed GPX4 degradation, indicating that inhibition of autophagy stabilizes GPX4 protein. Similarly, silencing BECN1 also prolonged GPX4 protein half-life under the same conditions (**Figure 5L and Supplementary Figure S5J**). Taken together, these results demonstrate that GPX4 is S-palmitoylated in macrophages, and this modification is essential for its lysosomal stability. Loss of palmitoylation promotes GPX4 polyubiquitination and degradation, underscoring S-palmitoylation as a key post-translational determinant of ferroptosis resistance in exosome-reprogrammed macrophages.

### 3.6 CaMK2A upregulates GPX4 through ZDHHC3-mediated inhibition of GPX4 degradation

**Figure 6:**
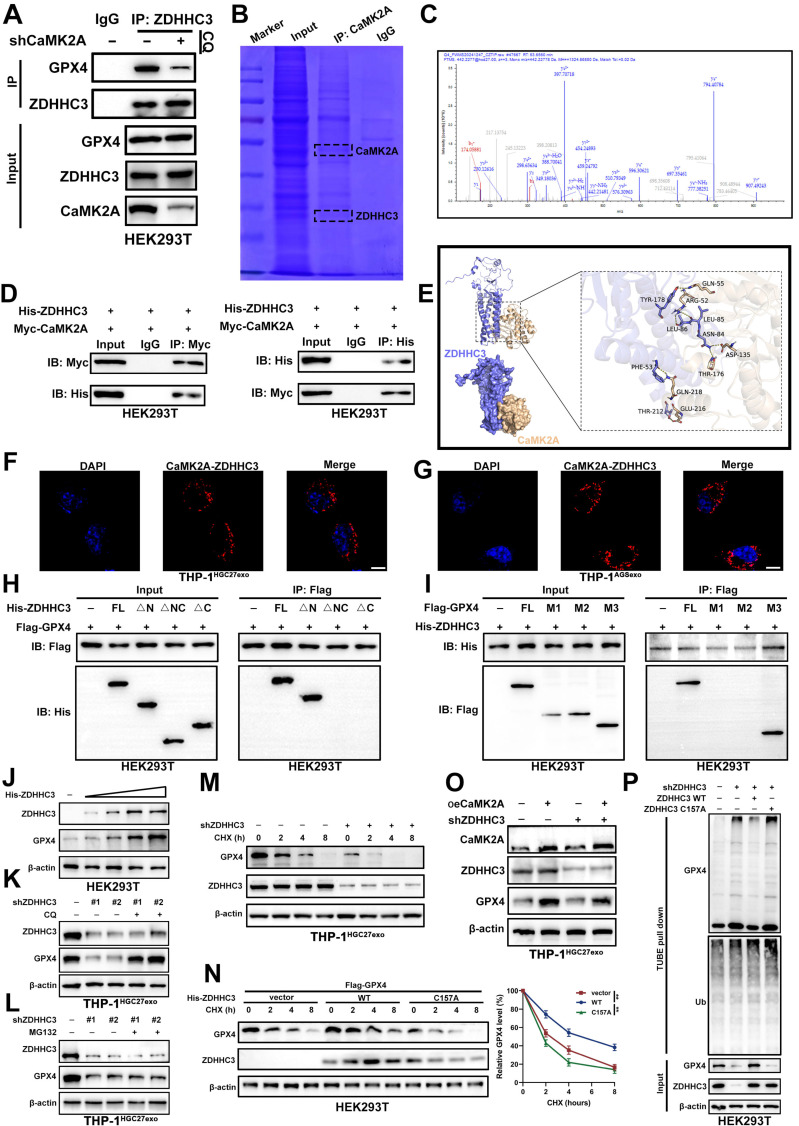
** CaMK2A upregulates GPX4 through ZDHHC3-mediated inhibition of GPX4 degradation. A.** Co**-**IP was performed to determine the interaction between ZDHHC3 and GPX4 under control or CaMK2A-silenced conditions. **B-C.** Co-IP assay and subsequent mass spectrometry analysis were used to determine ZDHHC3 as potential target of CaMK2A. **D.** Western blot was used to examine the interaction between CaMK2A and ZDHHC3. **E.** Structure-based molecular docking analysis was performed to detect the interaction between CaMK2A and ZDHHC3. **F-G.** Representative PLA images showing the interaction between endogenous CaMK2A and ZDHHC3 in THP-1 macrophages treated with HGC27-derived exosomes or AGS-derived exosomes. Scale bar: 10 μm. **H.** HEK293T cells transfected with Flag-GPX4 and indicated ZDHHC3 deletion constructs were subjected to Co-IP assay with Flag beads, followed by Western blot. **I.** HEK293T cells transfected with His-ZDHHC3 and indicated GPX4 deletion constructs were subjected to Co-IP assay with His beads, followed by Western blot. **J.** HEK293T cells transfected with Flag-GPX4 and increasing amounts of His-ZDHHC3 were subjected to Western blot analysis with indicated antibodies.** K.** THP-1 cells transfected with control or ZDHHC3 shRNA were subjected to Western blot analysis with indicated antibodies in the absence or presence of CQ. **L.** THP-1 cells transfected with control or ZDHHC3 shRNA were subjected to Western blot analysis with indicated antibodies in the absence or presence of MG132. **M.** Detecting GPX4 protein levels in THP-1 cells transfected as indicated and subsequently subjected to the CHX chase assay for indicated times. **N.** Detecting Flag-GPX4 protein levels in HEK293T cells transfected as indicated and subsequently subjected to the CHX chase assay for indicated times. **O.** Detecting GPX4 protein levels in TJP-1 cells transfected with oeCaMK2A or shZDHHC3 as indicated. **P.** Detecting GPX4 ubiquitination levels in HEK293T cells transfected as indicated and subsequently subjected to TUBE-pull down assay. Data are presented as mean ± SD of at least three independent experiments. *p < 0.05, **p < 0.01, ***p < 0.001. n.s., no significance.

Given that palmitoylation is catalyzed by palmitoyltransferases, we next sought to identify the enzyme responsible for GPX4 palmitoylation. To this end, we performed Co-IP followed by mass spectrometry analysis. By intersecting the differential proteins identified in the IP assay with the ZDHHC family of palmitoyltransferases, we narrowed the candidates to ZDHHC3, ZDHHC8, ZDHHC17, and ZDHHC20 (**Supplementary Figure S6A**). To determine which of these enzymes mediates GPX4 palmitoylation in macrophages following treatment with exosomal CaMK2A, we further performed IP assays and found that only ZDHHC3 displayed CaMK2A-dependent regulation of its interaction with GPX4 (**Figure 6A-C and Supplementary Figure S6B-D**). Reciprocal Co-IP confirmed this interaction, as CaMK2A co-precipitated with ZDHHC3 and vice versa (**Figure 6D**). Structural molecular docking further supported a stable interaction between CaMK2A and ZDHHC3 (**Figure 6E**). To further validate the interaction between CaMK2A and ZDHHC3 at the endogenous level, we performed PLA in exosome-educated THP-1 macrophages. Distinct PLA signals were readily detected in THP-1^HGC27exo^ and THP-1^AGSexo^ cells, indicating close spatial proximity between endogenous CaMK2A and ZDHHC3 in macrophages following GC-derived exosome treatment (**Figure 6F-G**).

To define the structural basis of the ZDHHC3-GPX4 interaction, we generated a series of His-tagged ZDHHC3 and Flag-tagged GPX4 truncation mutants (**Supplementary Figure S6E-F**). Co-IP mapping revealed that the C-terminal region of ZDHHC3 binds to the N-terminal domain of GPX4, establishing the interface essential for complex formation (**Figure 6H-I**).

Functionally, ZDHHC3 overexpression elevated GPX4 protein levels in a dose-dependent manner (**Figure 6J**), whereas ZDHHC3 knockdown significantly reduced GPX4 expression. This reduction was reversed by CQ, but not by MG132 (**Figure 6K-L**), indicating that ZDHHC3 regulates GPX4 stability primarily through the lysosomal degradation pathway. In CHX chase assays, ZDHHC3 depletion accelerated GPX4 turnover, while wild-type (WT) ZDHHC3, but not its catalytically inactive mutant (C157A), prolonged GPX4 half-life (**Figure 6M-N**).

Importantly, ZDHHC3 knockdown abolished the CaMK2A-induced upregulation of GPX4 (**Figure 6O**). Moreover, TUBE pull-down assays revealed that loss of ZDHHC3 markedly increased GPX4 polyubiquitination, whereas reintroduction of WT ZDHHC3, but not the C157A mutant, reversed this effect (**Figure 6P**). Flow cytometric analysis showed that silencing ZDHHC3 markedly reduced the proportion of CD68^+^CD206^+^ macrophages induced by GC-derived exosomes, whereas reintroduction of ZDHHC3 WT, but not ZDHHC3 C157A, restored this population (**Supplementary Figure S6G**). Consistently, qRT-PCR analysis demonstrated that the expression of M2-associated markers was significantly decreased upon ZDHHC3 knockdown, and this effect was rescued by ZDHHC3 WT but not by the C157A mutant (**Supplementary Figure S6H**). To conclude, these data identify ZDHHC3 as a key mediator linking CaMK2A to GPX4 stabilization. Exosomal CaMK2A binds and activates ZDHHC3, which in turn suppresses GPX4 polyubiquitination and lysosomal degradation, thereby maintaining GPX4 protein stability and promoting macrophage ferroptosis resistance.

### 3.7 ZDHHC3 catalyzes S-palmitoylation of GPX4 at C10

**Figure 7:**
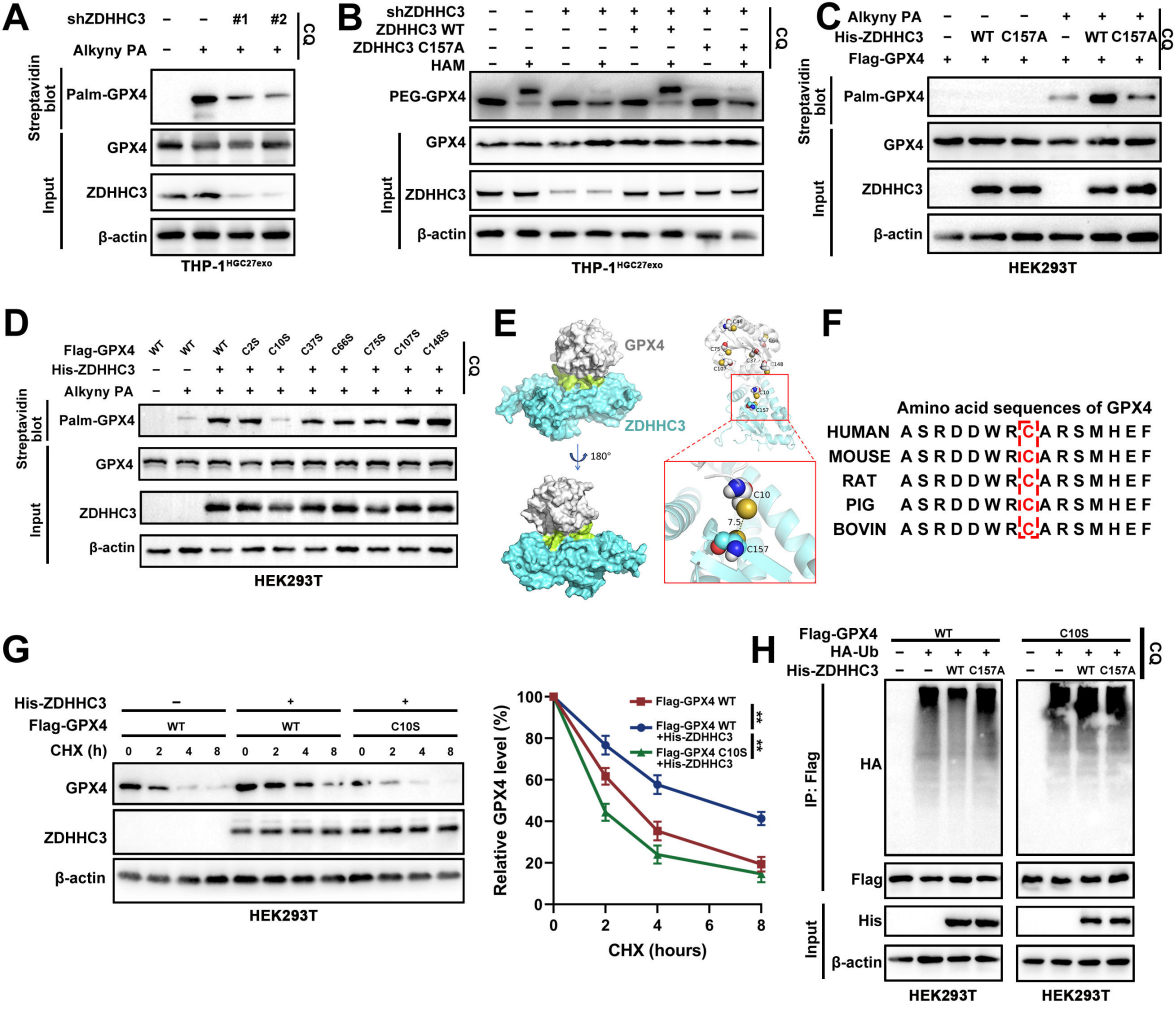
** ZDHHC3 catalyzes S-palmitoylation of GPX4 at C10. A.** THP-1 cells transfected as indicated were metabolically labeled with 50 μM Alkyny PA for 4 h by click reaction and subjected to streptavidin pulldown with the treatment of CQ, followed by Western blot using indicated antibodies. **B.** GPX4 palmitoylation in THP-1 cells transfected as indicated were analyzed by the APE assays upon CQ treatment. PEG-GPX4 bands indicated palmitoylated GPX4. **C-D.** HEK293T cells transfected as indicated were metabolically labeled with 50 μM Alkyny PA for 4 h by click reaction and subjected to streptavidin pulldown with the treatment of CQ, followed by Western blot. **E.** Structure-based molecular docking analysis was performed to detect the interaction between GPX4 and ZDHHC3. **F.** Amino acid sequencing of GPX4 in different species. **G.** Detecting Flag-GPX4 protein levels in HEK293T cells transfected as indicated and subsequently subjected to the CHX chase assay for indicated times. **H.** Detecting GPX4 ubiquitination levels in HEK293T cells transfected as indicated and subsequently subjected to d-IP. Data are presented as mean ± SD of at least three independent experiments. *p < 0.05, **p < 0.01, ***p < 0.001. n.s., no significance.

To determine whether ZDHHC3 directly mediates GPX4 S-palmitoylation, we first assessed GPX4 palmitoylation levels in THP-1 macrophages following ZDHHC3 knockdown. Loss of ZDHHC3 markedly reduced the incorporation of alkynyl palmitate into GPX4, indicating impaired palmitoylation (**Figure 7A**). Reintroduction of ZDHHC3 WT, but not ZDHHC3 C157A, restored GPX4 palmitoylation (**Figure 7B**). Consistently, overexpression of ZDHHC3 WT, but not ZDHHC3 C157A, significantly enhanced S-palmitoylation of Flag-GPX4 (**Figure 7C**).

Palmitoylation is a reversible PTM in which ZDHHC enzymes covalently attach C16 fatty acid chains to cysteine residues of substrate proteins. To identify the specific palmitoylation site on GPX4, we systematically mutated all seven cysteine residues to serine and co-transfected these constructs with His-ZDHHC3. Western blot analysis revealed that mutation of Cys10 (C10S) substantially diminished ZDHHC3-mediated GPX4 palmitoylation (**Figure 7D**). Structure-based molecular docking supported this finding, showing that the catalytic Cys157 of ZDHHC3 lies in close spatial proximity to GPX4 Cys10, suggesting a direct enzymatic interaction (**Figure 7E**). Moreover, sequence alignment demonstrated that the amino acid region surrounding Cys10 is highly conserved across species (**Figure 7F**).

Functionally, the GPX4 C10S mutant exhibited accelerated degradation kinetics and increased polyubiquitination relative to WT GPX4, mirroring the effects observed following pharmacologic palmitoylation inhibition (**Figure 7G-H**). In conclusion, these findings demonstrate that ZDHHC3 catalyzes GPX4 S-palmitoylation at the conserved Cys10 residue, a modification critical for maintaining GPX4 stability and preventing its lysosomal degradation in macrophages.

### 3.8 CaMK2A-mediated T176 phosphorylation of ZDHHC3 facilitates its interaction with GPX4

**Figure 8:**
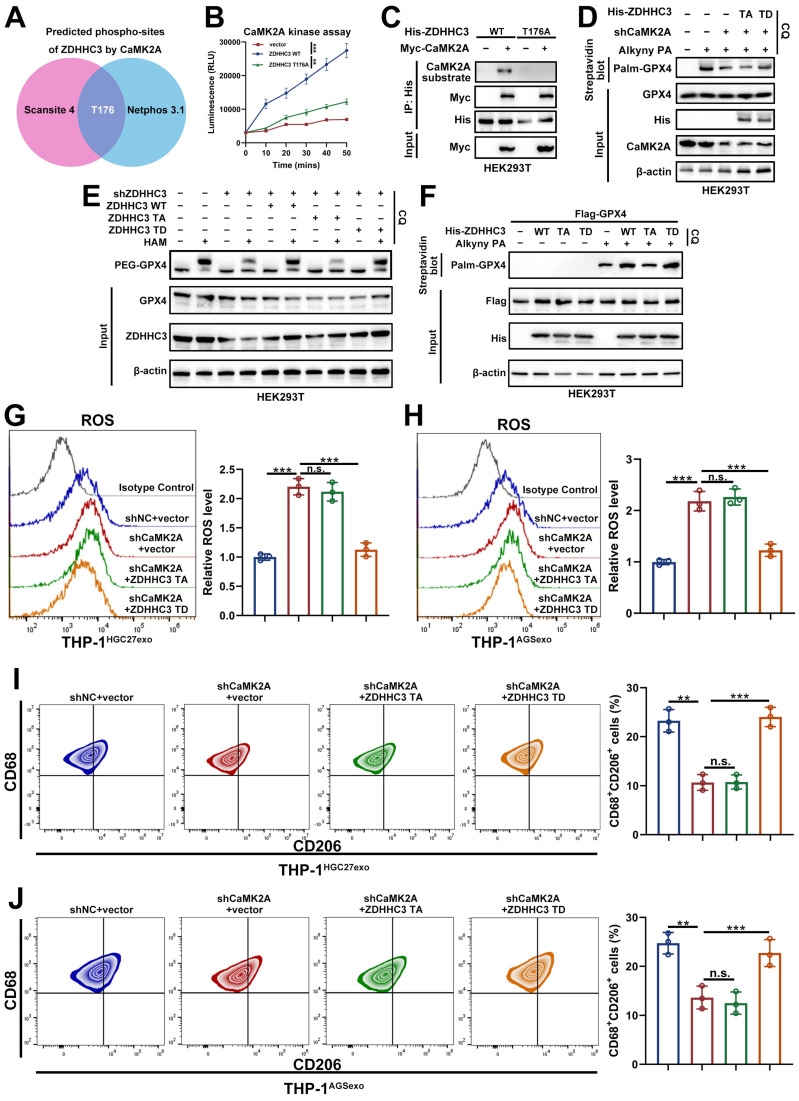
**CaMK2A-mediated T176 phosphorylation of ZDHHC3 facilitates its interaction with GPX4. A.** Two online phospho-sites prediction databases were used for prediction of potential ZDHHC3 phosphorylation residues by CaMK2A. **B.** Time-dependent CaMK2A kinase assay with ZDHHC3 WT or mutant protein precipitated from total cell lysate of HEK293T cells with exogenously overexpressed empty vector, ZDHHC3 WT, ZDHHC3 T176A, respectively. **C.** HEK293T cells transfected as indicated were subjected to Co-IP assay with His beads, followed by Western blot. **D.** HEK293T cells transfected as indicated were metabolically labeled with 50 μM Alkyny PA for 4 h by click reaction and subjected to streptavidin pulldown followed by Western blot using indicated antibodies. **E.** GPX4 palmitoylation in HEK293T cells transfected as indicated were analyzed by the APE assays in the absence or presence of HAM. PEG-GPX4 bands indicated palmitoylated GPX4. **F.** HEK293T cells transfected as indicated were metabolically labeled with PA for 4 h by click reaction and subjected to streptavidin pulldown followed by Western blot using indicated antibodies. **G-H.** Relative lipid ROS levels in THP-1 cells transfected as indicated determined by BODIPY C11 staining following FACS analysis. **I-J.** Flow cytometry was applied to measure CD68^+^CD206^+^ macrophages in THP-1 cells of each group. Data are presented as mean ± SD of at least three independent experiments. *p < 0.05, **p < 0.01, ***p < 0.001. n.s., no significance.

To delineate how CaMK2A regulates ZDHHC3-mediated GPX4 palmitoylation, we first predicted potential ZDHHC3 phosphorylation sites targeted by CaMK2A using two independent databases (NetPhos 3.1 and Scansite 4.0). Both algorithms consistently identified Thr176 (T176) as the only high-confidence phosphorylation site (**Figure 8A**).

To validate this prediction, an *in vitro* kinase assay was performed by incubating recombinant CaMK2A with purified WT or mutant ZDHHC3 proteins. CaMK2A exhibited a time-dependent increase in phosphorylation of WT ZDHHC3, whereas the phospho-deficient T176A mutant markedly attenuated this phosphorylation (**Figure 8B**). Likewise, overexpression of Myc-CaMK2A in cells enhanced phosphorylation of ZDHHC3 WT but not of the T176A mutant (**Figure 8C**), confirming that CaMK2A directly phosphorylates ZDHHC3 at T176.

We next examined whether this phosphorylation event modulates GPX4 S-palmitoylation. Silencing CaMK2A decreased GPX4 palmitoylation, which was rescued by the phosphomimetic ZDHHC3 T176D (TD) mutant but not by the non-phosphorylatable T176A (TA) mutant (**Figure 8D**). Similarly, depletion of ZDHHC3 reduced GPX4 S-palmitoylation, while re-expression of ZDHHC3 WT or TD, but not TA, restored the modification (**Figure 8E-F**).

Functionally, CaMK2A depletion led to increased ROS accumulation in THP-1 macrophages, indicative of ferroptosis induction. This effect was reversed by overexpression of ZDHHC3 TD but not TA (**Figure 8G-H**). Consistent trends were observed for ferroptosis-related indicators such as MDA and GSSG/GSH ratios (**Supplementary Figure S7A-D**). Furthermore, CaMK2A knockdown decreased both the proportion of CD68⁺CD206⁺ macrophages and expression of M2-associated markers, effects that were rescued by ZDHHC3 TD but not TA (**Figure 8I-J and Supplementary Figure S7E-F**).

To validate our findings in primary macrophages, we isolated bone BMDMs and examined whether the phenotype observed in THP-1-derived macrophages could be reproduced in this primary system. Consistent with our results in THP-1 cells, coculture with YTN16 cells markedly increased the GSH/GSSG ratio, reduced MDA levels, and suppressed ROS accumulation in BMDMs, indicating attenuation of ferroptosis-associated oxidative stress. In contrast, these effects were largely abolished when BMDMs were exposed to CaMK2A-silenced YTN16 cells, whereas treatment with Palmostatin B restored the phenotype (**Supplementary Figure S7G-I**). We next assessed macrophage polarization in BMDMs. Flow cytometric analysis showed that YTN16 cells significantly increased CD206 expression in BMDMs, whereas CaMK2A knockdown in YTN16 cells markedly weakened this effect; notably, Palmostatin B treatment restored CD206 expression (**Supplementary Figure S7J**). Consistently, qRT-PCR and Western blot revealed that the expression of M2-associated markers was elevated in YTN16-educated BMDMs, reduced upon CaMK2A silencing, and rescued by Palmostatin B. In parallel, GPX4 protein expression showed the same trend (**Supplementary Figure S7K-L**). Collectively, these findings demonstrate that CaMK2A-mediated phosphorylation of ZDHHC3 at Thr176 is indispensable for its interaction with GPX4, facilitating GPX4 S-palmitoylation, stabilization, and ferroptosis resistance in macrophages. This CaMK2A-ZDHHC3-GPX4 signaling axis thus serves as a pivotal mechanism driving M2 polarization and immune suppression within the GC microenvironment.

### 3.9 Targeted GPX4-mediated ferroptosis and protumoral polarization of macrophages suppresses GC and enhances the efficacy of the anti-PD-1/L1 response

**Figure 9:**
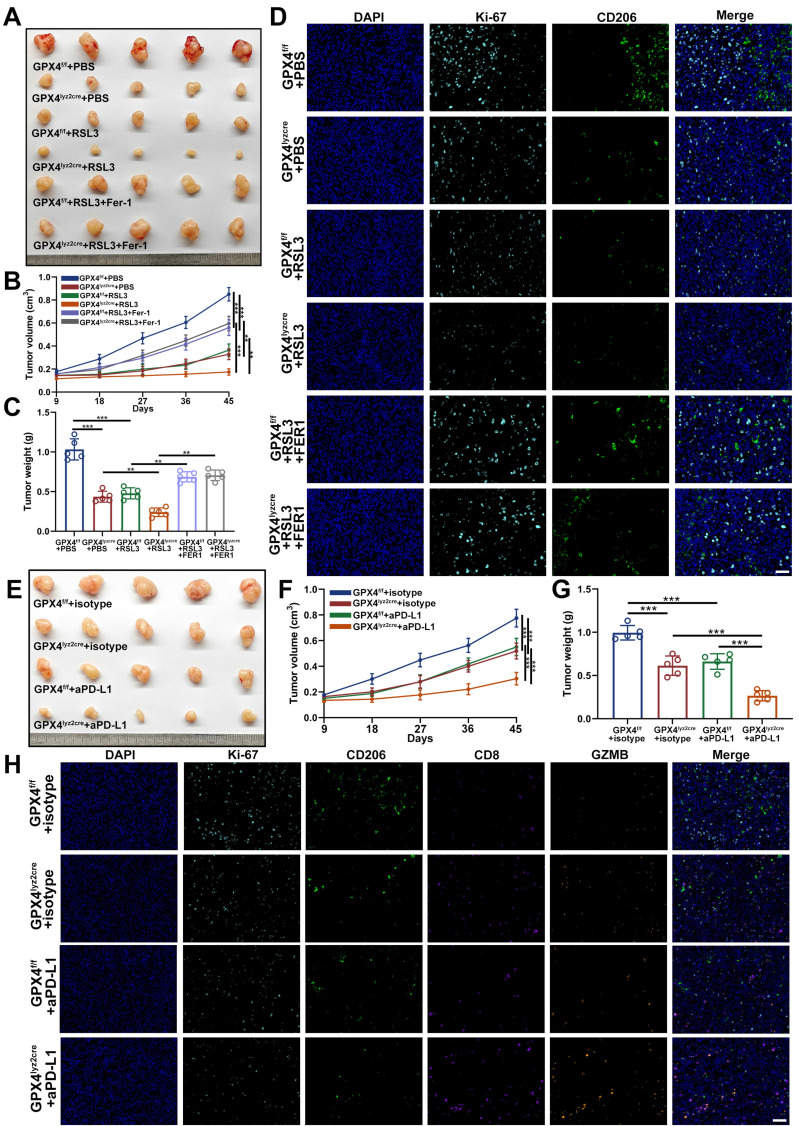
** Targeted GPX4-mediated ferroptosis and protumoral polarization of macrophages suppresses GC and enhances the efficacy of the anti-PD-1/L1 response. A.** The growth of YTN16 tumors in response to different treatments. Drug administration started at Day 9. RSL3:15 mg/kg, Fer-1: 10mg/kg. **B.** Differences in tumor volume in response to different treatments. **C.** Differences in tumor weight in response to different treatments. **D.** mIHC staining of Ki-67 and CD206 in tumors of different groups. **E.** The growth of YTN16 tumors in response to different treatments. **F.** Differences in tumor volume in response to different treatments. **G.** Differences in tumor weight in response to different treatments. **H.** mIHC staining of Ki-67, CD206, CD8 and PD-1 in tumors of different groups. Scale bar: 100 μm. Data are presented as mean ± SD of at least three independent experiments. *p < 0.05, **p < 0.01, ***p < 0.001. n.s., no significance.

To explore the functional interplay between GPX4 and TAMs *in vivo*, we evaluated the impact of macrophage-specific GPX4 deletion on GC progression. GPX4^lyz2cre^ mice and their GPX4^f/f^ littermate controls were implanted with YTN16 cells (**Supplementary Figure S8A**) and treated with PBS, the ferroptosis inducer RSL3, or RSL3 combined with Fer-1. Tumor growth was markedly reduced in GPX4^lyz2cre^ mice compared with GPX4^f/f^ controls, and this suppression was further enhanced by RSL3 treatment. Conversely, co-administration of Fer-1 partially reversed the inhibitory effect of GPX4 loss combined with RSL3, confirming the ferroptosis-dependent nature of the response (**Figure 9A-C**).

The mIHC revealed a pronounced decrease in Ki67⁺ proliferating cells and CD206⁺ macrophage infiltration within GPX4^lyz2cre^ tumors relative to GPX4^f/f^ controls. These reductions were further potentiated by RSL3 and partially rescued by Fer-1, reinforcing that macrophage ferroptosis contributes to tumor suppression (**Figure 9D**).

Given that TAMs critically modulate immune tolerance and resistance to immunotherapy, we next assessed whether macrophage GPX4-mediated ferroptosis influenced the efficacy of immune checkpoint blockade 52. Both macrophage-specific GPX4 knockout and anti-PD-L1 therapy independently inhibited tumor growth; notably, their combination achieved the most profound antitumor effect (**Figure 9E-G**). mIHC analyses demonstrated that either GPX4 ablation or PD-L1 blockade decreased Ki67⁺ and CD206⁺ cell populations while markedly increasing CD8⁺ T-cell infiltration and GZMB expression on CD8⁺ T cells. The combined treatment elicited the strongest activation of cytotoxic T-cell responses and tumor regression (**Figure 9H**).

To further strengthen the translational relevance of the CaMK2A-ZDHHC3-GPX4 axis, we next examined whether pharmacological targeting of this pathway could enhance the efficacy of immune checkpoint blockade *in vivo*. In the YTN16 syngeneic tumor model, treatment with the CaMK2A inhibitor KN93 (inhibitor of CaMK2A) alone moderately suppressed tumor growth, whereas anti-PD-L1 monotherapy also produced an inhibitory effect. Notably, the combination of KN93 and anti-PD-L1 showed the most pronounced antitumor activity, as evidenced by reduced tumor size, slower tumor growth kinetics, and lower tumor weight compared with either treatment alone (**Supplementary Figure S8B-D**). We further evaluated whether pharmacological inhibition of palmitoylation could similarly improve immunotherapy response. Treatment with 2-BP alone led to partial tumor suppression, while its combination with anti-PD-L1 resulted in a markedly greater reduction in tumor volume and tumor weight than either single treatment (**Supplementary Figure S8E-G**). Taken together, these findings demonstrate that targeting macrophage GPX4 enhances ferroptosis, disrupts protumoral polarization, and is associated with reinvigorated antitumor immunity. Importantly, blockade of the CaMK2A-ZDHHC3-GPX4 axis can sensitize tumors to PD-1/PD-L1 blockade *in vivo*, further supporting the therapeutic potential of targeting this signaling pathway in GC.

### 3.10 Clinical relevance and functional implications of GPX4, especially TAM-derived GPX4 in GC

**Figure 10:**
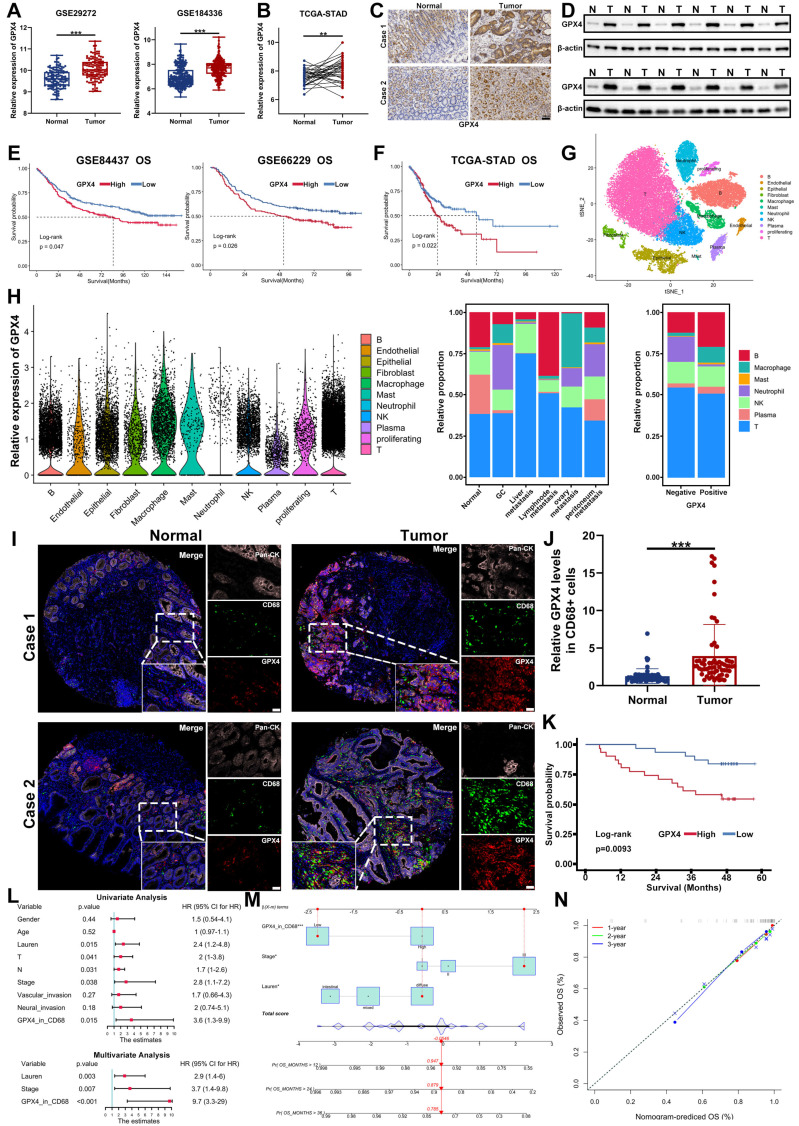
** Clinical relevance and functional implications of GPX4, especially TAM-derived GPX4 in GC. A-B.** Expression of GPX4 in GC tissues and normal tissues in GSE29272, GSE184336 and TCGA-STAD. **C.** IHC staining was used to detect the expression of GPX4 in GC tissues and normal tissue. **D.** Western blot was carried out to measure the expression of GPX4 in GC tissues and normal tissue. **E-F.** Survival analysis showing the predictive value of GPX4 in GSE84437, GSE66229 and TCGA-STAD cohort. **G.** The immune cellular landscape of GC (GSE163558). **H.** Expression of GPX4 in immune cells and the distribution proportion of immune cells in different tissues. **I-J.** IF staining showing the proportion of infiltrating CD68^+^ cells and GPX4 expression in CD68^+^ cells in tumor tissues compared to normal tissues. Scale bar: 20 μm. **K.** Survival analysis indicating a significant survival advantage of GC patients with low GPX4 expression in CD68^+^ cells. **L.** Univariate and multivariate Cox analysis of OS. **M-N.** A nomogram was constructed based on the independent prognostic factors of GC. Data are presented as mean ± SD of at least three independent experiments. *p < 0.05, **p < 0.01, ***p < 0.001. n.s., no significance.

To delineate the clinical relevance of GPX4, particularly TAMs-derived GPX4, in GC, we analyzed multiple independent transcriptomic datasets, including GSE29272, GSE184336, and TCGA. Across all cohorts, GPX4 expression was significantly elevated in GC tissues compared with adjacent normal tissues (**Figure 10A-B**). These observations were further validated in our own patient cohort by IHC and Western blot, which consistently demonstrated upregulated GPX4 expression in GC specimens relative to matched non-tumorous tissues (**Figure 10C-D**).

Survival analyses based on GSE84437, GSE66229, and TCGA datasets revealed that high GPX4 expression correlated with worse overall survival, whereas low GPX4 levels predicted favorable clinical outcomes (**Figure 10E-F**). To further characterize the immune landscape of GC, we analyzed single-cell RNA sequencing data (GSE163558), which enabled high-resolution mapping of GPX4 expression across immune subsets. GPX4 was found to be predominantly expressed in TAMs, where its expression strongly correlated with enhanced macrophage infiltration (**Figure 10G-H**).

IF staining of tissue microarrays corroborated these findings, revealing prominent colocalization of GPX4 with CD68⁺ macrophages in GC tissues (**Figure 10I-J**). Clinically, patients exhibiting low GPX4 expression in CD68⁺ macrophages displayed significantly prolonged survival compared with those with high expression (**Figure 10K**). Multivariate analysis further identified GPX4 expression in CD68⁺ macrophages as an independent prognostic factor for GC, outperforming conventional clinicopathological parameters (**Figure 10L**).

Based on these results, we established a prognostic nomogram integrating GPX4 expression and clinical variables to quantitatively predict patient outcomes. Calibration curves at 1, 2, and 3 years demonstrated excellent agreement between predicted and observed survival rates (**Figure 10M-N**), and ROC analyses confirmed that the nomogram exhibited superior predictive accuracy compared with individual factors (**Supplementary Figure S8A-C**). Collectively, these data underscore the clinical and functional significance of GPX4 in GC. In particular, TAMs-enriched GPX4 emerges as a robust and independent prognostic biomarker, offering strong potential for patient stratification and therapeutic guidance in GC immunotherapy.

## 4. Discussion

In this study, we elucidated a novel mechanism by which GC-derived exosomal CaMK2A modulates macrophage function to promote tumor progression. Our findings reveal that exosomal CaMK2A is internalized by macrophages, where it phosphorylates the palmitoyltransferase ZDHHC3. This phosphorylation event enhances the palmitoylation of GPX4 at C10, thereby stabilizing the protein by preventing its lysosomal degradation. The stabilized GPX4 inhibits ferroptosis in macrophages and drives their polarization towards an M2 phenotype, thereby contributing to the establishment of an immunosuppressive microenvironment that promotes the advancement of GC.

Given its intricate and distinct molecular pathogenesis, coupled with a complex TME, the progression of GC occurs at a rapid pace, severely limiting the efficacy of conventional treatment options. Furthermore, this complexity significantly hampers the effectiveness of emerging therapeutic strategies, including targeted therapy and immunotherapy 9, 53, 54. Accounting for the main component of immune cell infiltration in the TME, TAMs can constitute almost 50% of the tumor mass and is featured by functional and phenotypic plasticity, which plays crucial roles in the occurrence and progression of GC 40. Macrophages exhibit distinct roles in tumor biology, with M1 macrophages generally exerting anti-tumor effects, while M2 macrophages promote tumor progression through immune suppression and tissue remodeling 13, 55. In the context of GC, TAMs have been linked to poor prognosis, with their M2 polarization contributing to immune evasion and metastasis 56. Our results extend previous studies that have highlighted the role of exosomes as mediators of tumor-immune communication. While tumor-derived exosomes have long been recognized as drivers of immune suppression, the specific protein cargoes responsible for this process have not been fully elucidated 57, 58. Our findings uncover a mechanism by which GC cells utilize exosomal CaMK2A to reprogram TAMs towards the M2 phenotype, thereby establishing a mechanistic connection between tumor-derived exosomes and the functional modulation of immune cells.

A pivotal finding of our study is the identification of protein palmitoylation as a crucial regulator of macrophage function within the TME. Palmitoylation, a reversible lipid modification, has traditionally been associated with processes such as membrane trafficking and protein stability in various cellular contexts 34, 35, 59, 60. Although GPX4 has been reported to be palmitoylated by ZDHHC8 and ZDHHC20 49, 50, its involvement in immune cells, particularly in the regulation of ferroptosis, remains underexplored. Our data demonstrate that the phosphorylation of ZDHHC3 by CaMK2A facilitates the palmitoylation of GPX4 at C10. This modification prevents the degradation of GPX4, thereby stabilizing its expression and enabling macrophages to resist ferroptosis. Consequently, our study establishes a novel link between palmitoylation and ferroptosis regulation, offering new insights into how lipid modifications govern cell fate within the immune microenvironment.

The involvement of GPX4 in macrophages is crucial for understanding ferroptosis regulation within the TME. As an important regulator of ferroptosis in tumors, GPX4 not only modulates tumor growth but also impacts tumor immune microenvironment 44, 45. Recent studies have highlighted that the inhibition of GPX4 expression in triple-negative breast cancer promotes ferroptosis in tumor cells, concomitantly enhancing the infiltration of various T cell subsets, including CD4^+^ and CD8^+^ T cells. This shift transforms the TME from a "cold" to a "hot" state 61. Nonetheless, the exact role of GPX4 in immune cell function remains insufficiently elucidated. GPX4-mediated ferroptosis and phenotypic polarization of TAMs profoundly alter the TME of GC, thereby promoting tumor progression. These findings underscore the potential therapeutic value of targeting macrophage-derived GPX4 in GC treatment, highlighting the significance of our investigation. Previous studies have demonstrated that TAMs can express PD-L1, with its expression correlating with the immunosuppressive activity of these macrophages and facilitating tumor immune evasion 62. Moreover, other research has suggested that reduced GPX4 expression in colorectal adenocarcinoma compromises the efficacy of PD-1 blockade, potentially through the activation of the cGAS-STING pathway, which drives immune cell infiltration 63. We demonstrated that the specific knockout of GPX4 in macrophages, in combination with anti-PD-L1 therapy, achieved significantly enhanced antitumor efficacy compared to either GPX4 knockout in macrophages or anti-PD-L1 treatment alone. Upon further investigation of the clinical and functional profiles of GPX4 expression in GC, we observed that GPX4 expression was significantly elevated in tumor tissues compared to normal counterparts, suggesting that GPX4 serves as a potential and critical oncogenic factor in GC. Furthermore, the expression of GPX4 in both tumors and CD68^+^ macrophages was strongly associated with the prognosis of GC patients, providing a robust and reliable prognostic tool for patient outcome prediction.

However, our current data do not distinguish whether this immune remodeling is driven by ferroptosis-associated signals, altered cytokine/chemokine secretion, or both. Likewise, although GPX4 loss was associated with enhanced ferroptotic stress and reduced M2-like features, these two processes should not be interpreted as a simple linear upstream-downstream relationship. We therefore interpret these findings cautiously and propose that macrophage GPX4 loss coordinately affects ferroptotic status and protumoral polarization, thereby creating a microenvironment more permissive for cytotoxic T-cell infiltration and activation. Further studies are needed to clarify the relative contribution of these mechanisms. Another potential limitation of the experiments is that GPX4^fl/fl^ mice, rather than Lyz2^cre^-only mice, were used as controls. Although our findings are consistent with a tumor-promoting role of macrophage-associated GPX4, the potential influence of the Lyz2^cre^ background itself on myeloid cell behavior and antitumor immunity cannot be completely excluded and should be addressed in future studies with additional control cohorts.

In summary, this study uncovers a novel signaling axis wherein GC-derived exosomal CaMK2A modulates protumoeal polarization and ferroptosis inhibition in macrophages through phosphorylation-dependent palmitoylation of GPX4. Furthermore, the specific knockout of GPX4 in macrophages, in combination with anti-PD-L1 therapy, resulted in significantly enhanced antitumor efficacy. These findings not only advance our understanding of tumor-immune interactions but also highlight a promising therapeutic target for GC. By targeting this pathway, it may be possible to reverse M2 polarization, restore ferroptosis in macrophages, and reprogram the TME into a more anti-tumorigenic state.

## Supplementary Material

Supplementary figures and tables.

## Figures and Tables

**Figure 11:**
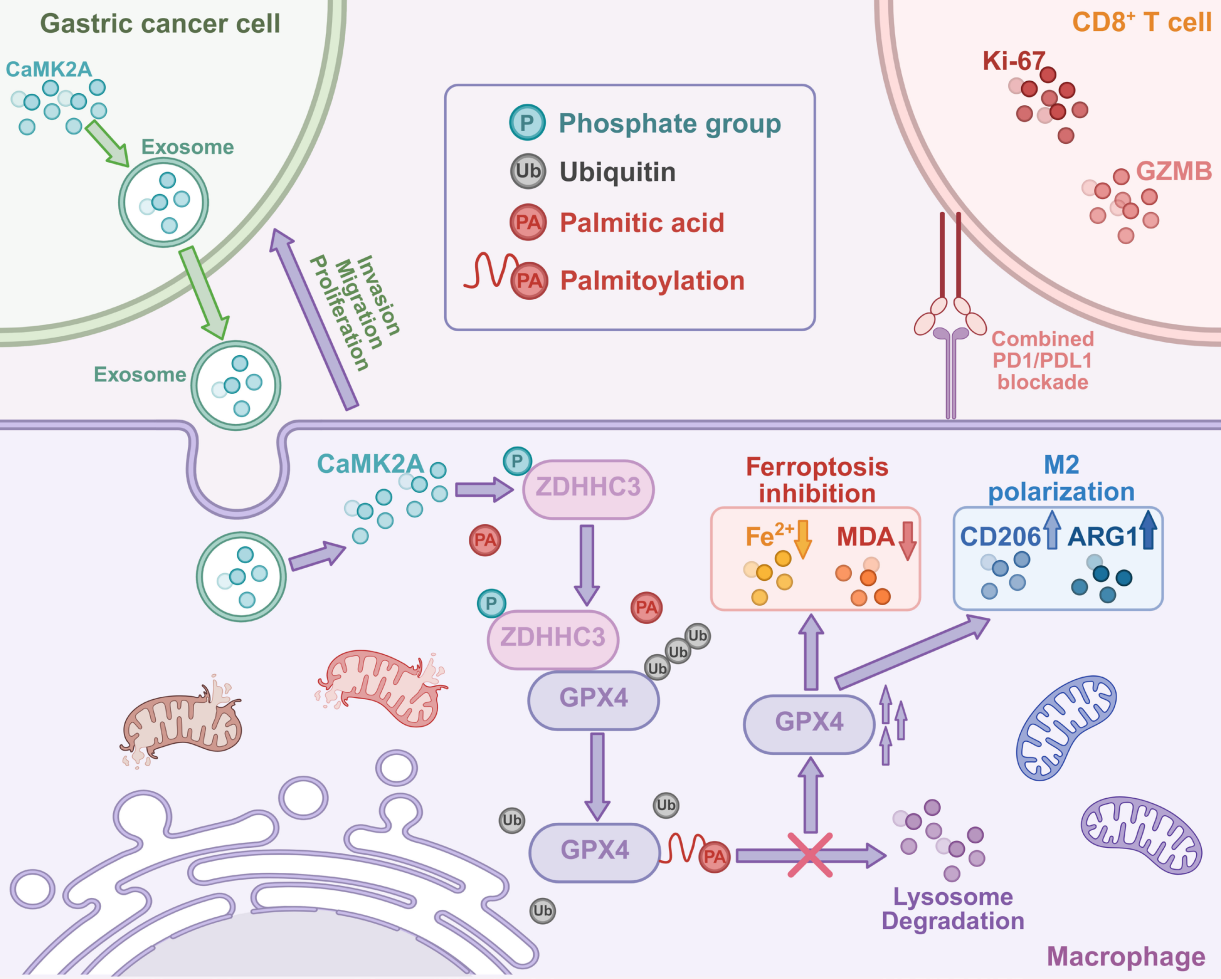
** Schematic illustration of this study.** GC-derived exosomal CaMK2A induces protumoral polarization and ferroptosis inhibition of macrophages via phosphorylation-dependent S-palmitoylation of GPX4 (the figure was created in https://BioRender.com).

## Data Availability

The datasets used and/or analyzed in this study are available upon reasonable request from the corresponding author.
