# Supplementary material for: Targeting the exosomal CaMK2A-ZDHHC3-GPX4 pathway reprograms tumor-associated macrophages and enhances anti-PD-1/PD-L1 immunotherapy in gastric cancer
Source: Int J Biol Sci. 2026 May 18;22(11):5659–84. doi: 10.7150/ijbs.128976 (PMC13282743; doi:10.7150/ijbs.128976)
Supplement: Supplementary file 1 — Supplementary figures and tables. [file ijbsv22p5659s1.pdf]

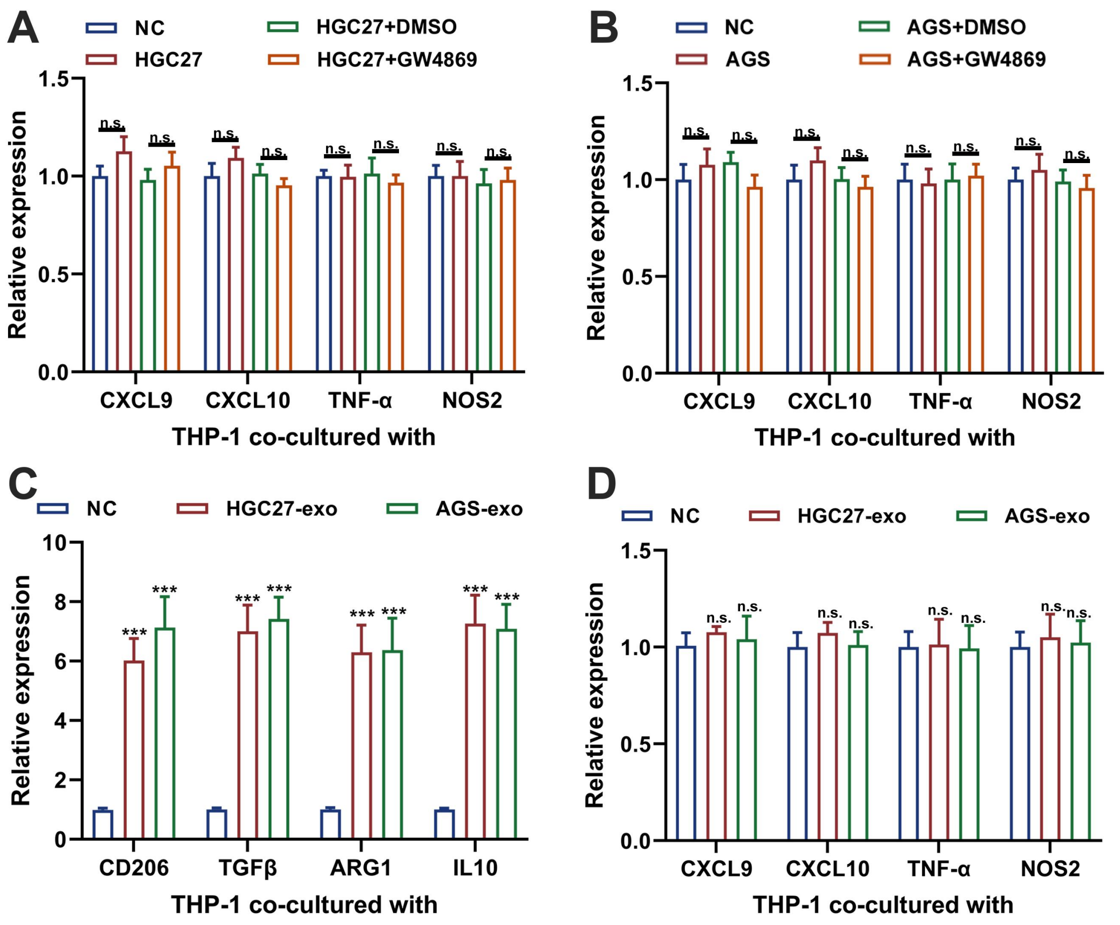

Supplementary Figure. S1 GC-derived exosomal CaMK2A promotes M2 polarization of macrophages

A-B. qRT-PCR was utilized to detect the levels of M1 markers (CXCL9, CXCL10, TNF-α and NOS2) in THP-1 cells with or without the co-culture with GC cells co-culture and the treatment of GW4869 on GC cells. **C.** qRT-PCR was adopted to measure the levels of M2 markers in THP-1 cells in each group with or without GC cell-derived exosome treatment. **D.** qRT-PCR was carried out to observe the levels of M1 markers in THP-1 cells in each group with or without GC cell-derived exosome treatment. Data are presented as mean ± SD of at least three independent experiments. *$p < 0.05$, **$p < 0.01$, ***$p < 0.001$. n.s., no significance.

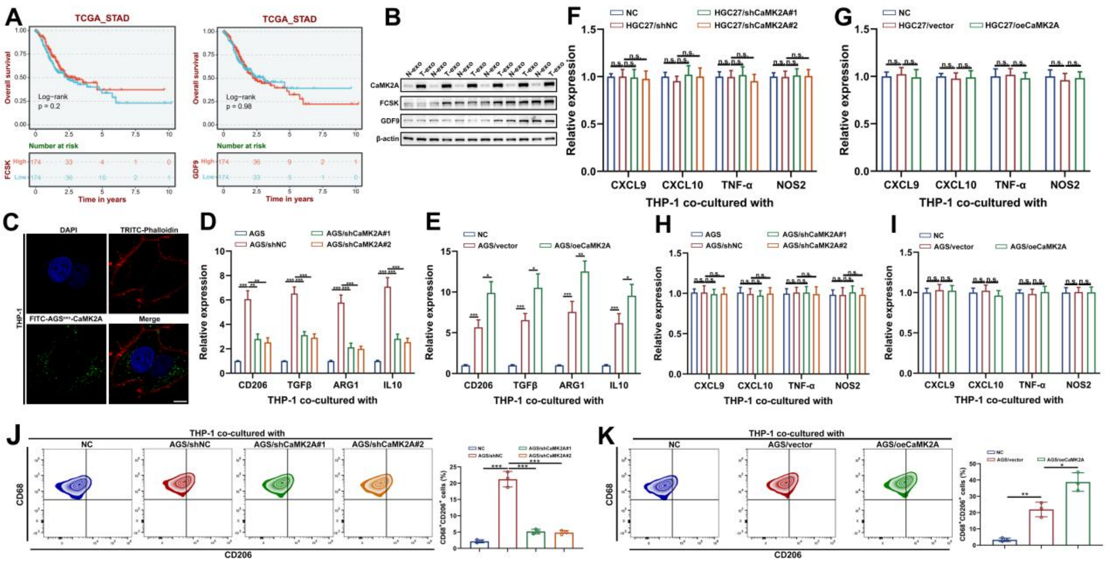

Supplementary Figure. S2 GC-derived exosomal CaMK2A promotes M2 polarization of macrophages

A. Survival analysis of FCSK and GDF9 in TCGA-STAD cohort. **B.** Western blot was used to detect the expression levels of CaMK2A in GC tissues-derived exosomes and corresponding normal tissues-derived exosomes. **C.** Fluorescence microscopy images illustrated the process of AGS cells-derived exosomal CaMK2A transmitted to macrophages. AGS cells-derived CaMK2A were labeled by FITC (green). TRITC-Phalloidin (red) was adopted for labeling cytoskeleton of THP-1 cells. Scale bar: 10 μm. **D-E.** qRT-PCR was adopted to measure the levels of M2 markers in THP-1 cells co-cultured with AGS cells transfected with shCaMK2A, oeCaMK2A, or untransfected. **F-I.** qRT-PCR was adopted to measure the levels of M1 markers in THP-1 cells co-cultured with GC cells transfected with shCaMK2A, oeCaMK2A, or untransfected. **J-K.** Flow cytometry was applied to measure CD68$^+$CD206$^+$ macrophages in THP-1 cells co-cultured with AGS cells, either transfected with shCaMK2A, oeCaMK2A, or untransfected. Data are presented as mean ± SD of at least three independent experiments. **p* < 0.05, ***p* < 0.01, ****p* < 0.001. n.s., no significance.

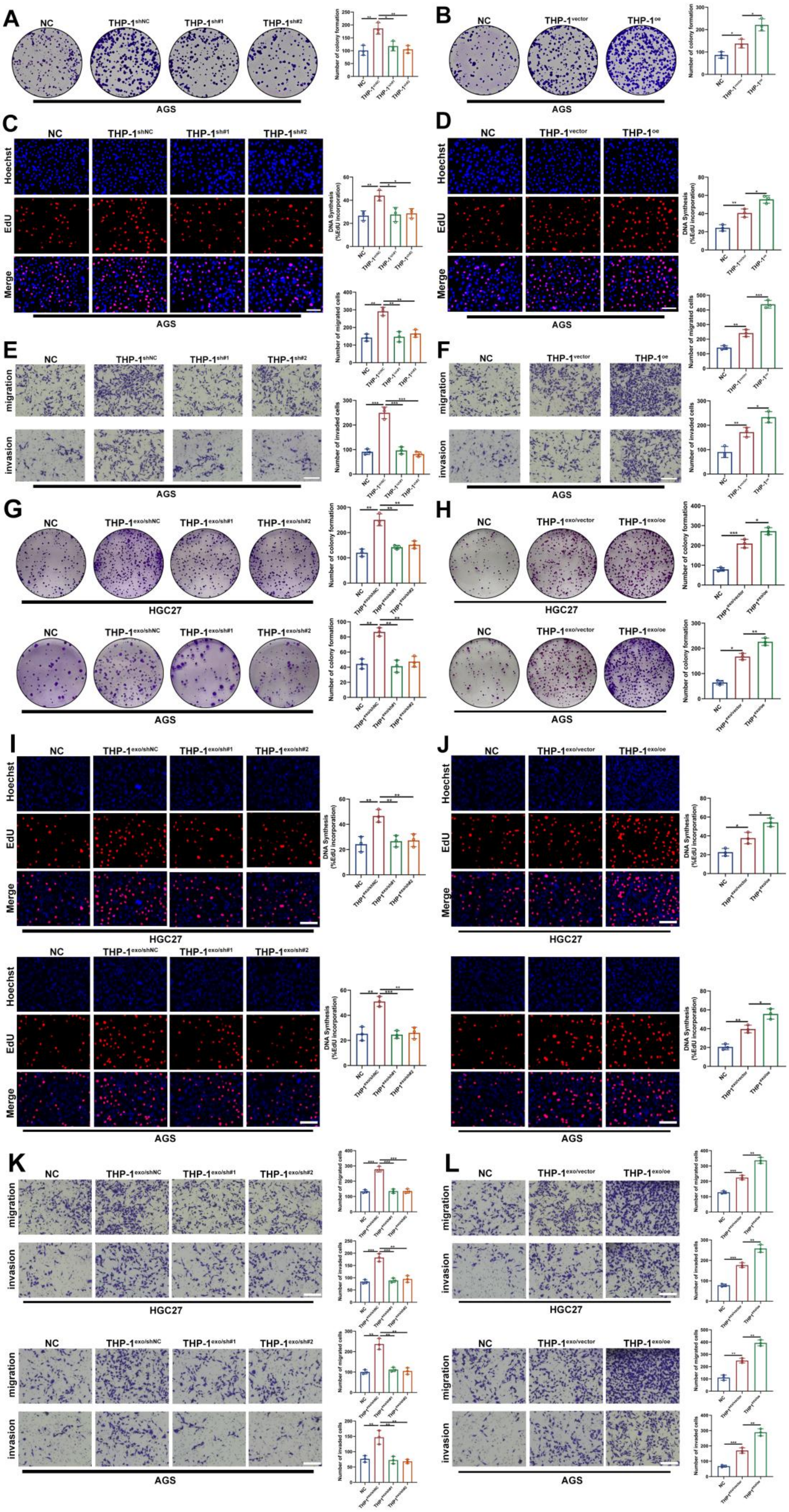
A
NC
THP-1shNC
THP-1sh#1
THP-1sh#2
AGS
Number of colony formation
B
THP-1vector
THP-1oe
C
Hoechst
EdU
Merge
DNA Synthesis (%EdU incorporation)
D
Number of migrated cells
E
migration
invasion
Number of invaded cells
F
G
THP-1exo/shNC
THP-1exo/sh#1
THP-1exo/sh#2
HGC27
H
THP-1exo/vector
THP-1exo/oe
I
J
K
L

Supplementary Figure. S3 Exosomal CaMK2A treated macrophages in turn enhances malignant behaviors of GC cells

A-B. Colony formation assay was used to determine the effects of THP-1 cells on the proliferation of AGS cells. THP-1 cells were pre-cocultured with GC cells transfected with CaMK2A overexpressing or silencing. **C-D.** EdU assay was utilized to explore the influence of pre-treated THP-1 cells on the proliferation of AGS cells. Scale bar: 100 μm. **E-F.** Transwell assay was performed to detect the effects of pre-treated THP-1 cells on the migration and invasion of AGS cells. Scale bar: 100 μm. **G-H.** Colony formation assay was used to determine the effects of exosomes-treated THP-1 cells on the proliferation of GC cells. **I-J.** EdU assay was utilized to explore the influence of exosomes-treated THP-1 cells on the proliferation of GC cells. Scale bar: 100 μm. **K-L.** Transwell assay was performed to detect the effects of exosomes-treated THP-1 cells on the migration and invasion of GC cells. Scale bar: 100 μm. Data are presented as mean ± SD of at least three independent experiments. *$p < 0.05$, **$p < 0.01$, ***$p < 0.001$. n.s., no significance.

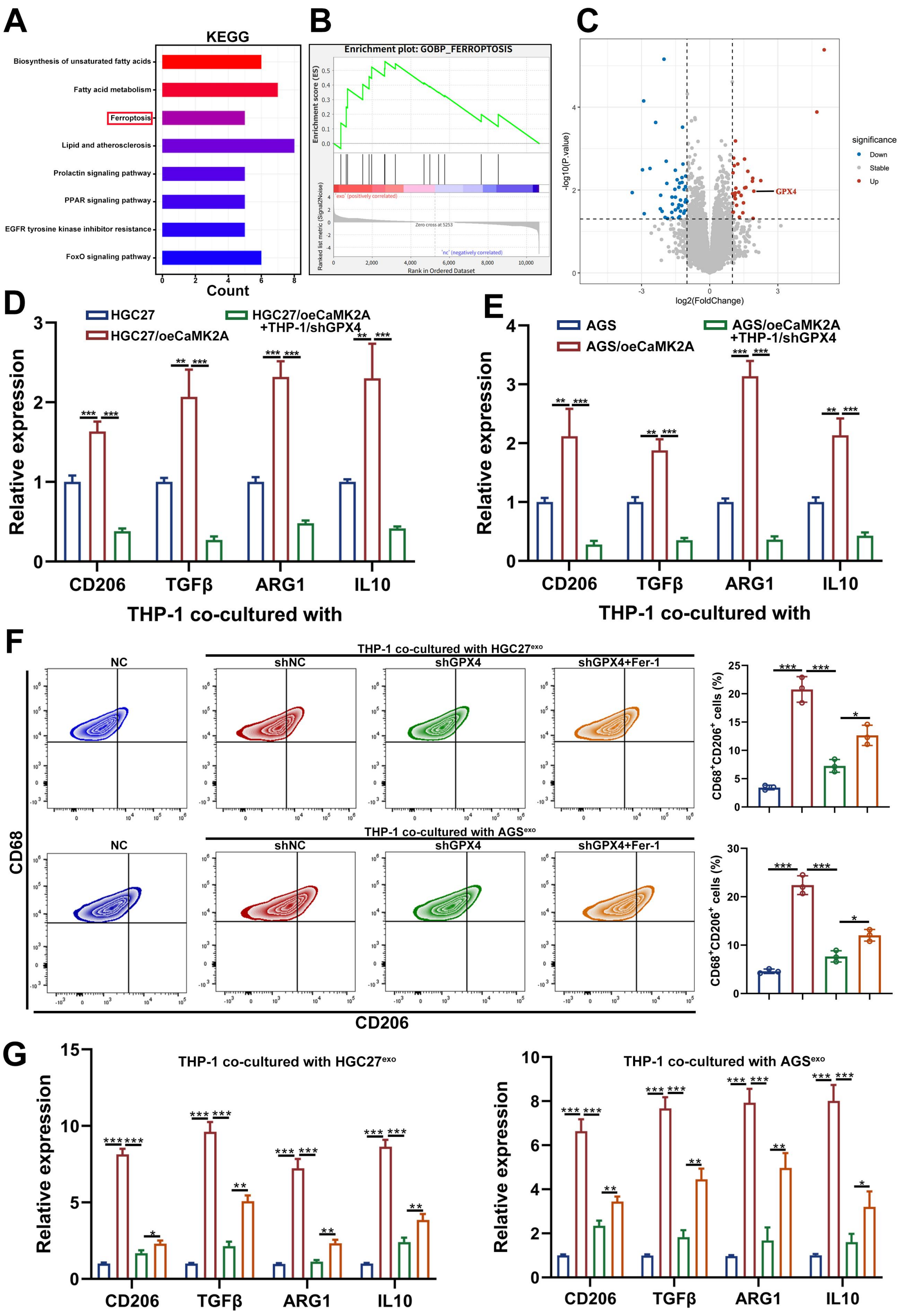

Supplementary Figure. S4 Exosomal CaMK2A upregulates GPX4, thus inhibiting ferroptosis and M2 polarization in macrophages

A. KEGG analysis revealed that differentially expressed proteins are associated with the ferroptosis pathway. **B.** GO enrichment analysis demonstrated that differentially expressed

proteins are associated with the ferroptosis pathway. **C.** Proteomic profiling of THP-1 cells incubated with exosomes derived from either HGC27 cells or control cells. **D-E.** qRT-PCR was adopted to measure the levels of M2 markers in THP-1 cells of each group. **F.** Flow cytometry was applied to measure $CD68^{+}CD206^{+}$ macrophages in THP-1 cells treated with HGC27-derived exosomes or AGS-derived exosomes and transfected with shZDHHC3, followed by re-expression of ZDHHC3 WT or the catalytically inactive mutant ZDHHC3 C157A. **G.** qRT-PCR was adopted to measure the levels of M2 markers in THP-1 cells of each group. Data are presented as mean ± SD of at least three independent experiments. $^{*}p < 0.05$, $^{**}p < 0.01$, $^{***}p < 0.001$. n.s., no significance.

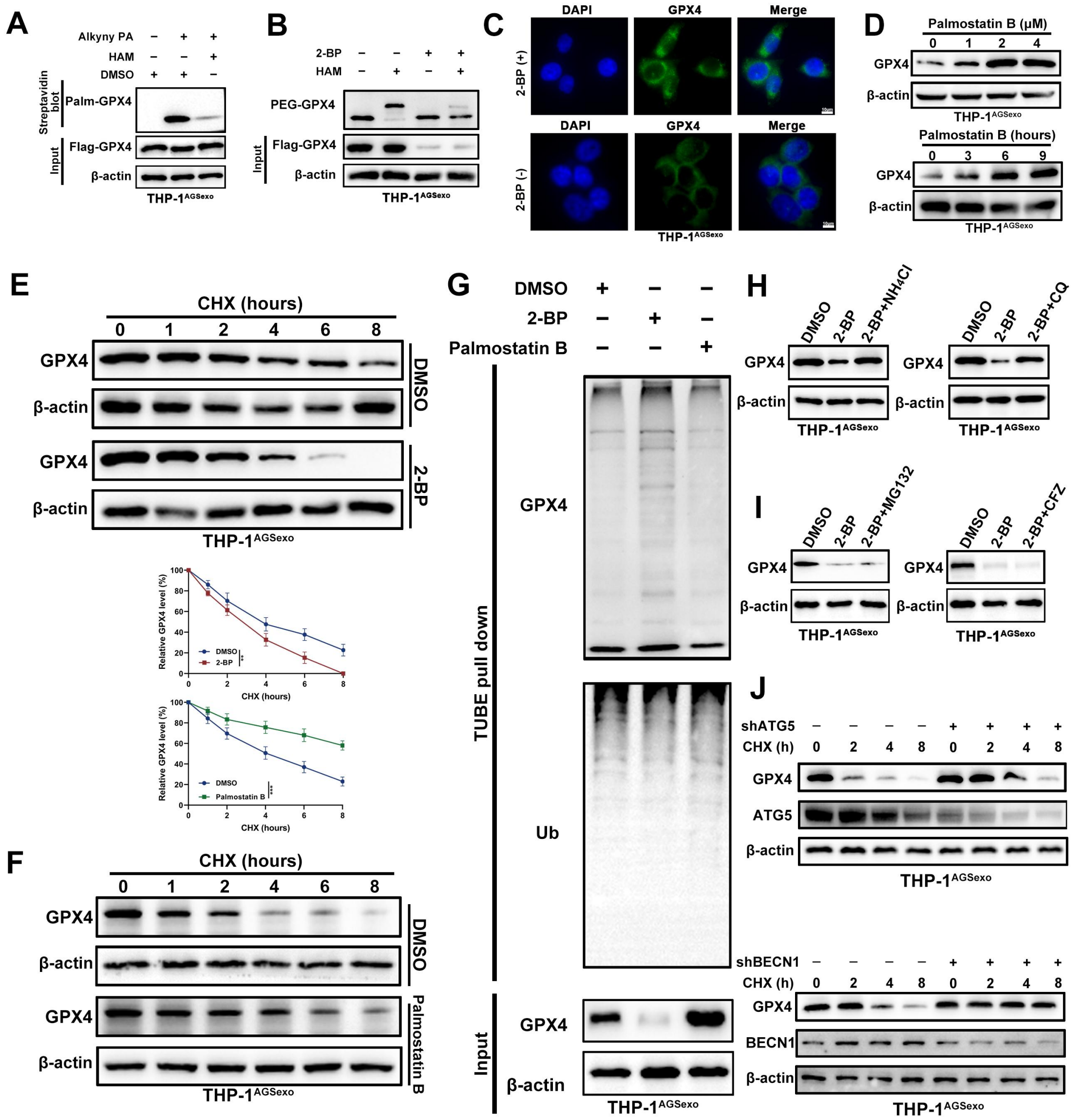

Supplementary Figure. S5 S-palmitoylation of GPX4 enhances its protein stability via the lysosomal pathway

A. THP-1 cells transfected with Flag-GPX4 were metabolically labeled with 50 μM Alkyny PA for 4 h by click reaction and subjected to streptavidin pulldown with or without the treatment of HAM, followed by Western blot using indicated antibodies. **B.** GPX4 palmitoylation in THP-1 cells were analyzed by the APE assays, upon 2-BP treatment (50 μM) in the absence or presence of HAM. PEG-GPX4 bands indicated palmitoylated GPX4. **C.** THP-1 cells were subjected to confocal analysis in the absence or presence of 2-BP. Scare bar: 10 μm. **D.** Western blot was used for detection of GPX4 protein levels in THP-1 cells treated as indicated amount and time of Palmostatin B. **E-F.** Detecting GPX4 protein levels in THP-1 cells treated with 50 μM 2-BP (H) or 2 μM palmostatin B (I) for 24 h and subsequently subjected to the CHX chase assay for indicated

times. **G.** Detecting GPX4 ubiquitination levels in THP-1 cells treated with 2 μM palmostatin B for 24 h and subsequently subjected to TUBE-pull down assay. **H-I.** Western blot analysis for GPX4 protein levels in THP-1 cells treated as indicated. Cells were treated with 2-BP (50 μM), MG132 (20 mM), Carfilzomib (100 nM), NH_4Cl (20 mM), or CQ (100 mM) for 5 h before collection. **J.** Cells were transduced with control shRNA or shRNA targeting ATG5 or BECN1, followed by CHX chase analysis at the indicated time points. Western blot was performed to detect the degradation of GPX4. Data are presented as mean ± SD of at least three independent experiments. *$p < 0.05$, **$p < 0.01$, ***$p < 0.001$. n.s., no significance.

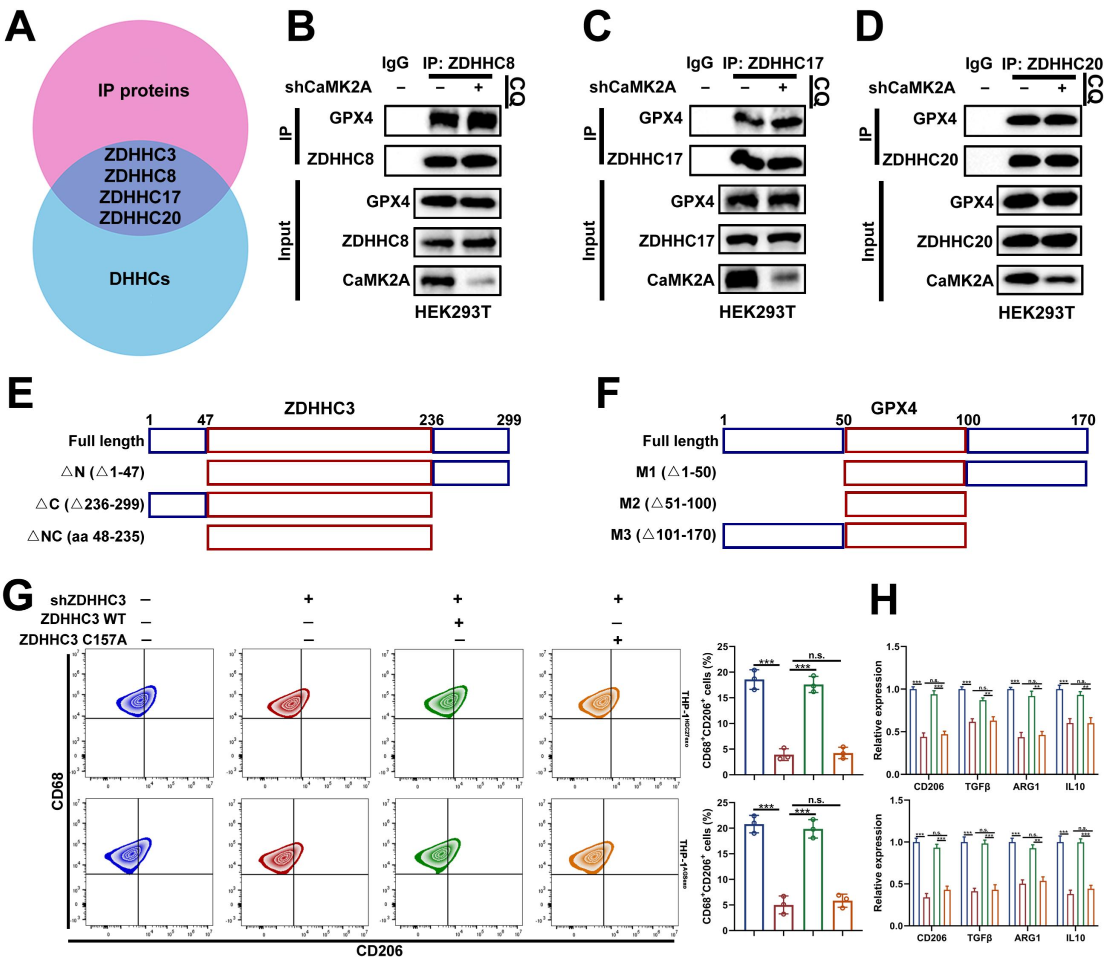

Supplementary Figure. S6 ZDHHC3 is the major palmitoyltransferase linking exosomal CaMK2A to GPX4 palmitoylation and macrophage polarization

A. Venn diagram showing the overlap between proteins identified by Co-IP/mass spectrometry and members of the ZDHHC palmitoyltransferase family, yielding four candidate enzymes: ZDHHC3, ZDHHC8, ZDHHC17, and ZDHHC20. **B-D.** Co-IP assays in HEK293T cells examining the interaction of GPX4 with candidate ZDHHC family members under control or CaMK2A-silenced conditions. **E.** Schematic illustration of full-length and truncation constructs of ZDHHC3 used for domain-mapping analysis. **F.** Schematic illustration of full-length and truncation constructs of GPX4 used for interaction mapping. **G.** Representative flow cytometry plots and quantification of $CD68^{+}CD206^{+}$ macrophages in THP-1 cells treated with HGC27- or AGS-derived exosomes following ZDHHC3 knockdown and re-expression of ZDHHC3 WT or ZDHHC3 C157A. **H.** qRT-PCR analysis of M2-associated markers under the indicated conditions. Data are presented as mean ± SD of at least three independent experiments. *$p < 0.05$, **$p < 0.01$, ***$p < 0.001$. n.s., no significance.

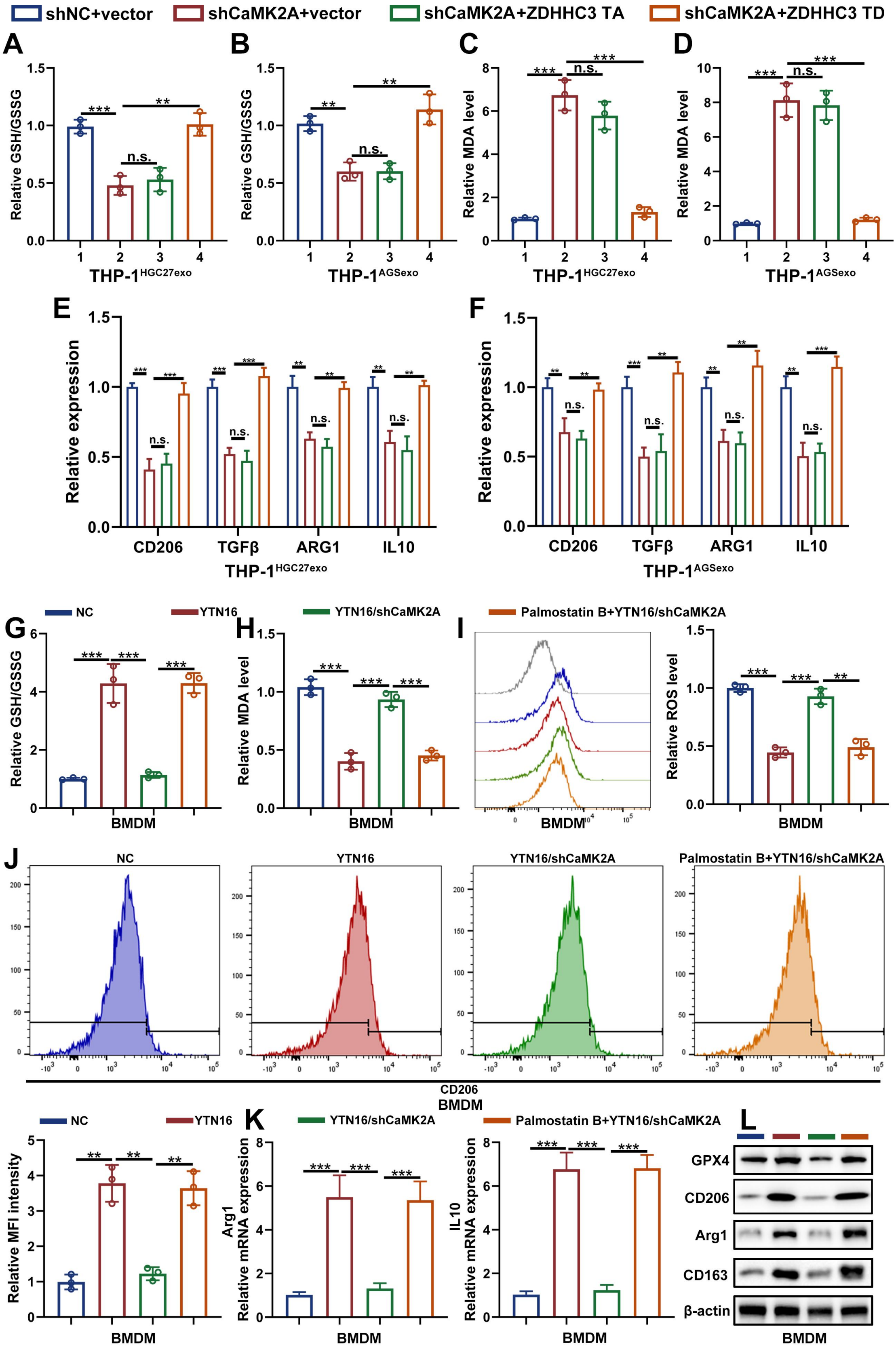

Supplementary Figure. S7 CaMK2A-mediated T176 phosphorylation of ZDHHC3 facilitates

its interaction with GPX4

A-D. Levels of GSH/GSSG ratio and MDA in THP-1 cells of each group. **E-F.** qRT-PCR was adopted to measure the levels of M2 markers in THP-1 cells of each group. **G-I.** Relative GSH/GSSG ratio, MDA level, and ROS accumulation in BMDMs of each group. **J.** Representative flow cytometry histograms and quantification of CD206 expression in BMDMs of each group. **K.** qRT-PCR analysis of Arg1 and IL10 expression in BMDMs of each group. **L.** Western blot analysis of GPX4 and M2-associated markers in BMDMs of each group. Data are presented as mean ± SD of at least three independent experiments. *$p < 0.05$, **$p < 0.01$, ***$p < 0.001$. n.s., no significance.

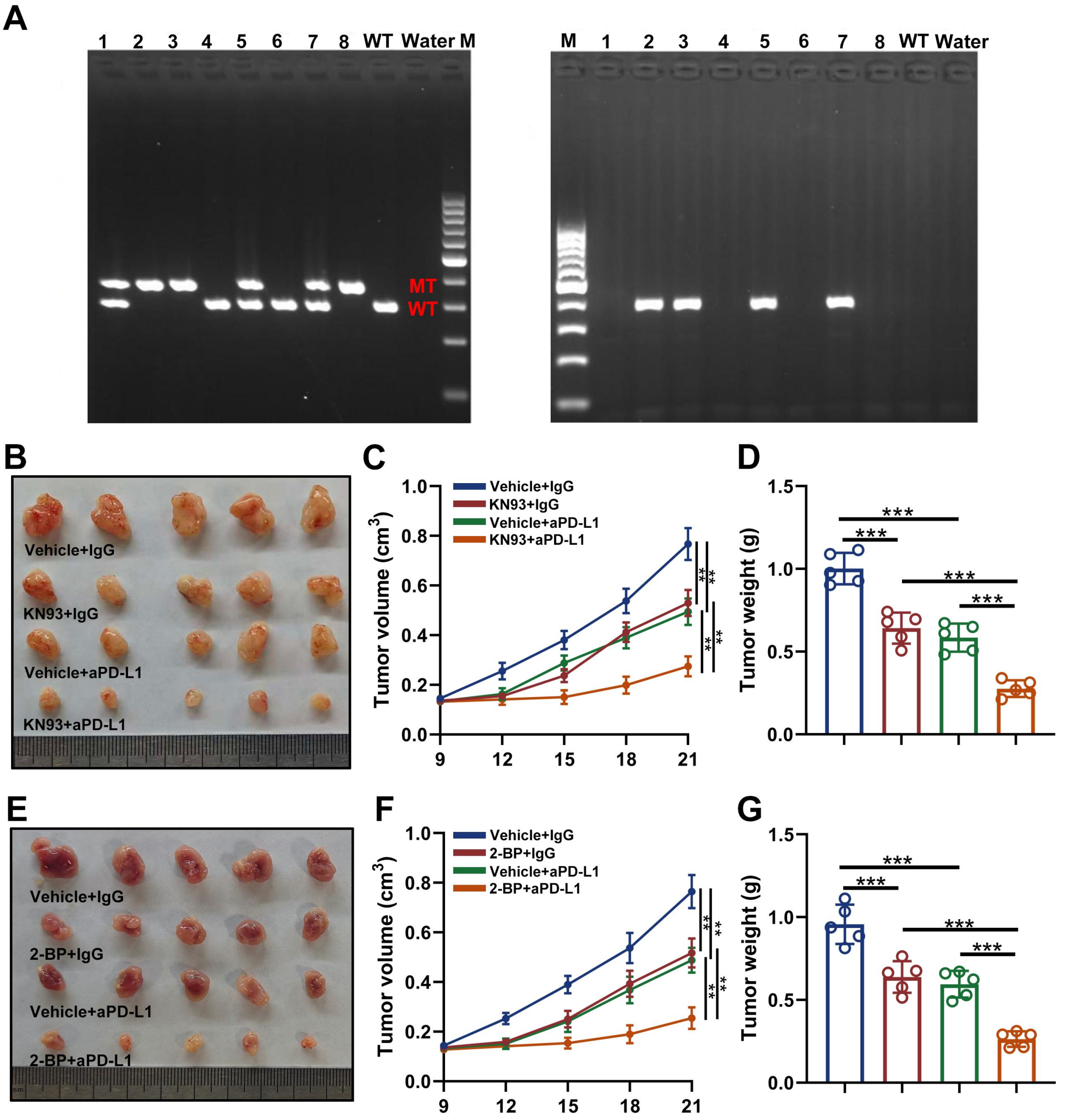

Supplementary Figure. S8 Pharmacological targeting of the CaMK2A-ZDHHC3-GPX4 axis enhances the antitumor efficacy of anti-PD-L1 therapy in vivo..

A. Genotyping analysis showing the confirmation of the $GPX4^{LysMcre}$ mice. MT was used as positive control. **B.** Representative images of tumors from the YTN16 syngeneic tumor model treated with vehicle + IgG, KN93 + IgG, vehicle + anti-PD-L1, or KN93 + anti-PD-L1. **C.** Tumor growth curves of mice in the indicated treatment groups. KN93: 1mg/kg. **D.** Tumor weights at the experimental endpoint. **E.** Representative images of tumors from mice treated with vehicle + IgG, 2-BP + IgG, vehicle + anti-PD-L1, or 2-BP + anti-PD-L1. 2-BP: 40mg/kg. **F.** Tumor growth curves of mice in the indicated treatment groups. **G.** Tumor weights at the experimental endpoint. Data are presented as mean ± SD of at least three independent experiments. *$p < 0.05$, **$p < 0.01$, ***$p < 0.001$. n.s., no significance.

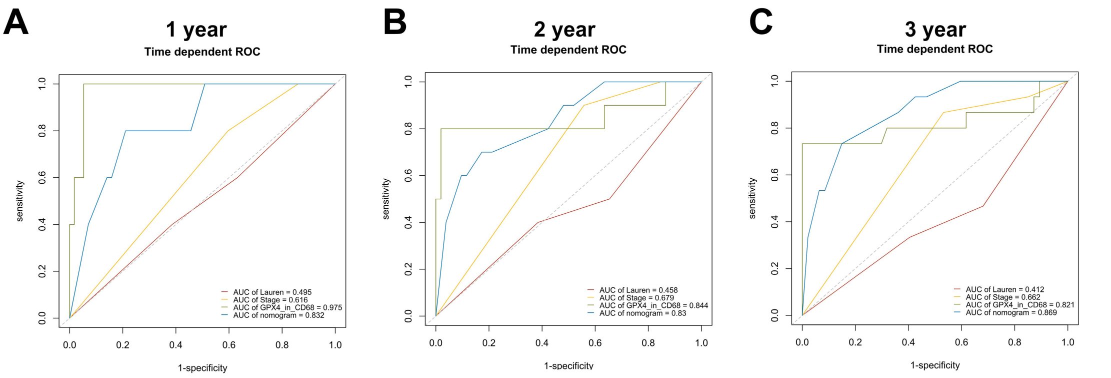

Supplementary Figure. S9 Clinical relevance and functional implications of GPX4, especially TAM-derived GPX4 in GC

A-C. ROC analysis revealed that the predictive performance of the nomogram surpassed that of any individual prognostic factor

Supplementary Table. 1 Sequences of primers used in this study

**Primer sequence**	
β-actin	Forward: 5'-ATTGCCGACAGGATGCAGAA-3'<br>Reverse: 5'-GCTGATCCACATCTGCTGGAA-3'
CD206	Forward: 5′-ATCACGAAGCCAAGGTCCAG-3′<br>Reverse: 5′-GTGGGTGAACCGAACCTCTT-3′
TGFβ	Forward: 5'-ATGGTGGAAACCCACAACGA-3'<br>Reverse: 5'-GTTCAGGTACCGCTTCTCGG-3'
ARG1	Forward: 5'-GGGTTGACTGACTGGAGAGC-3'<br>Reverse: 5'-CTGTAGGGCCTTCTTCCACC-3'
IL10	Forward: 5'-CTGTTGCCTGGTCCTCCTG-3'<br>Reverse: 5'-TTCTCAGCTTGGGGCATCAC-3'
CXCL9	Forward: 5'-GATTGGTGCCCAGTTAGCCT-3'<br>Reverse: 5'-CCACCGGACAGCACTCTAAA-3'
CXCL10	Forward: 5'-GTGGCATTCAAGGAGTACCTC-3'<br>Reverse: 5'-TGATGGCCTTCGATTCTGGATT-3'
TNFα	Forward: 5'-AGAACTCACTGGGGCCTACA-3'<br>Reverse: 5'-GCTCCGTGTCTCAAGGAAGT-3'
NOS2	Forward: 5'-TTCAGTATCACAACCTCAGCAAG-3'<br>Reverse: 5'-TGGACCTGCAAGTTAAAATCCC-3'
CaMK2A	Forward: 5'-AAGCAGCAGTGAGGGAGATG-3'<br>Reverse: 5'-AGGGTGAGGAAGATGAAGGA-3'
GPX4	Forward: 5'-GCCTTTGCCGCCTACTGA-3'<br>Reverse: 5'-TGTGCCCGTCGATGTCCT-3'
**Genotyping primers**	
Tg-Lyz2-iCre	Forward: 5'-GCATCGCATTGTCTGAGTAGGTG-3'<br>Reverse: 5'-GGGAACAACCATGACAGTGTATGC-3'
GPX4 loxp	Forward: 5'-GAGTTCCTGGGCTTGGTGTG-3'<br>Reverse: 5'-GGCTGAGAATTCGTGCATGG-3'

Supplementary Table. 2 Antibodies used in this study

Western blot

**Primary antibody**		
β-actin	CST	#4967
HSP70	Abcam	Ab181606
CD9	Abcam	Ab263019
CD81	Abcam	Ab109201
Calnexin	Abcam	Ab133615
CaMK2A	CST	#19945
FCSK	Proteintech	13541-1-AP
GDF9	Abcam	29309-1-AP
Anti-Flag	Abcam	Ab205606
Anti-His	Abcam	ab18184
Anti-Myc	Abcam	Ab32
GPX4	Abcam	Ab125066
ATG5	Abcam	Ab109327
BECN1	Abcam	Ab207612
ZDHHC3	Abcam	Ab31837
ZDHHC8	Proteintech	27179-1-AP
ZDHHC17	Abcam	Ab253017
ZDHHC20	Invitrogen	PA5-101778
Ub	CST	#20326
**Secondary antibody**		
Anti-rabbit IgG	Cell signaling Technology	#7074
Anti-mouse IgG	Cell signaling Technology	#7076

IHC and IF

**Primary antibody**		
CD68	Abcam	Ab303566
CD206	Abcam	Ab64693
Ki-67	Abcam	Ab15580
CD8	Abcam	Ab217344
GZMB	Abcam	Ab208586
Pan-CK	Proteintech	26411-1-AP
**Secondary antibody**		
Goat Anti-Mouse IgG H&L<br>(Alexa Fluor® 647)	Abcam	Ab150115
Goat Anti-Rabbit IgG H&L<br>(Alexa Fluor® 594)	Abcam	Ab150080

FC

Primary antibody		
CD68	Biolegend	333806
CD206	Biolegend	321104
